# The CPEB Protein Orb2 Has Multiple Functions during Spermatogenesis in *Drosophila melanogaster*

**DOI:** 10.1371/journal.pgen.1003079

**Published:** 2012-11-29

**Authors:** Shuwa Xu, Nathaniel Hafer, Blessing Agunwamba, Paul Schedl

**Affiliations:** 1 Department of Biology, California Institute of Technology, Pasadena, California, United States of America; 2 UMass Center for Clinical and Translational Science, University of Massachusetts Medical School, Worcester, Massachusetts, United States of America; 3 Memorial Sloan-Kettering Cancer Center, New York, New York, United States of America; 4 Department of Molecular Biology, Princeton University, Princeton, New Jersey, United States of America; Harvard Medical School, Howard Hughes Medical Institute, United States of America

## Abstract

Cytoplasmic Polyadenylation Element Binding (CPEB) proteins are translational regulators that can either activate or repress translation depending on the target mRNA and the specific biological context. There are two CPEB subfamilies and most animals have one or more genes from each. *Drosophila* has a single CPEB gene, *orb* and *orb2*, from each subfamily. *orb* expression is only detected at high levels in the germline and has critical functions in oogenesis but not spermatogenesis. By contrast, *orb2* is broadly expressed in the soma; and previous studies have revealed important functions in asymmetric cell division, viability, motor function, learning, and memory. Here we show that *orb2* is also expressed in the adult male germline and that it has essential functions in programming the progression of spermatogenesis from meiosis through differentiation. Like the translational regulators *boule (bol)* and *off-schedule (ofs)*, *orb2* is required for meiosis and *orb2* mutant spermatocytes undergo a prolonged arrest during the meiotic G2-M transition. However, *orb2* differs from *boule* and *off-schedule* in that this arrest occurs at a later step in meiotic progression after the synthesis of the meiotic regulator *twine*. *orb2* is also required for the orderly differentiation of the spermatids after meiosis is complete. The differentiation defects in *orb2* mutants include abnormal elongation of the spermatid flagellar axonemes, a failure in individualization and improper post-meiotic gene expression. Amongst the *orb2* differentiation targets are *orb* and two other mRNAs, which are transcribed post-meiotically and localized to the tip of the flagellar axonemes. Additionally, analysis of a partial loss of function *orb2* mutant suggests that the *orb2* differentiation phenotypes are independent of the earlier arrest in meiosis.

## Author Summary

Cytoplasmic Polyadenylation Element Binding (CPEB) proteins bind and recognize CPE sequences in the 3′ UTRs of target mRNAs and can activate and/or repress their translation depending on the mRNA species and the biological context. *Drosophila* has two CPEB family genes, *orb* and *orb2*. *orb* is expressed in the germline of both sexes and has critical functions at multiple steps during oogenesis; however, it plays only a limited role in spermatogenesis. Here we show that the second CPEB family gene *orb2* has the opposite sex specificity in germline development. While it appears to be dispensable for oogenesis, *orb2* has essential functions during spermatogenesis. It is required for programming the orderly and sequential progression of spermatogenesis from meiosis through differentiation. *orb2* mutants fail to execute the meiotic G2-M transition and exhibit a range of defects in the process of sperm differentiation.

## Introduction

Proteins in the Cytoplasmic Polyadenylation Element Binding (CPEB) family were first identified in *Drosophila* ovaries and *Xenopus* oocytes [1]–[4]. In both organisms the CPEB proteins function in the localization and translational regulation of mRNAs encoding key developmental and polarity determinants as well as factors controlling the process of egg maturation. Since then CPEB family proteins have been implicated in many other biological contexts. These include translational regulation of embryonic cell division [5], [6], regulation of p53 expression [7], [8], synaptic plasticity in the rat hippocampus [9], long-term memory in *Aplysia* [10], [11] and spermatogenesis in the worm [12]. The CPEB proteins bind to CPE elements in the 3′ UTRs of target mRNAs and can both repress and activate translation. Translation activation typically involves the phosphorylation of the CPEB protein and the subsequent recruitment of a cytoplasmic poly A polymerase which extends the poly A tail [13].

Most animals have two or more CPEB genes. Completed genome sequences reveal that humans, mice, and *C. elegans* have four genes, while there are only two CPEBs, *orb* and *orb2*, in *Drosophila*. The homology between the CPEBs is largely restricted to the C-terminal region of the protein, where two RNA-Recognition Motif (RRM) domains are found, while the N-terminal domain is highly divergent even amongst closely related species. Phylogenetic trees indicate that the CPEB genes fall into two different subgroups. One subgroup includes *Drosophila orb*, mouse CPEB1 and the canonical *Xenopus* CPEB, while the other subgroup contains the second *Drosophila* CPEB gene, *orb2*, as well as mammalian CPEB 2, 3, and 4 [12], [14].

The *Drosophila orb* gene has been extensively characterized. Its expression appears to be restricted to the germline as neither mRNA nor protein can be detected in somatic tissues of the embryo, larvae and adult. While a male-specific Orb isoform is expressed in the male germline, its activity is not absolutely essential since the fertility of *orb* null males is reduced but not eliminated (Agunwamba and Xu, unpublished). In contrast, *orb* plays a central role in the process of oogenesis. *orb* expression is first activated during the mitotic divisions that ultimately generate an egg chamber containing 15 nurse cells and an oocyte. At this stage *orb* activity is required for the proper specification of the oocyte. Subsequently, *orb* is required for establishing the anterior-posterior and dorsal-ventral axes of the egg and embryo. Amongst the key *orb* mRNA regulatory targets are the polarity determinants *oskar* and *gurken* [15]–[18].

Unlike *orb*, the second *Drosophila* CPEB gene, *orb2*, is broadly expressed in both the soma and germline. The highest levels of Orb2 are in the embryonic, larval and adult CNS, and in the germ cells of the male testes [19]. There are two Orb2 isoforms, one of 75 kD and the other of 60 kD. The larger isoform is expressed in somatic tissues and the germline of both sexes, while the smaller isoform is found in testes but is not detected elsewhere. The isoforms share a 542 C-terminal amino acid sequence, but have unique N-termini of 162 and 9 amino acids respectively. Included in the common region is the conserved C-terminal CPEB signature RRM type RNA binding and zinc finger domains. The N-terminal half of both isoforms has short conserved sequences rich in serine or histidine interspersed with poorly conserved sequences containing poly-glutamine or poly-glycine repeats [19].

As might be expected from its broad expression pattern, *orb2* has a number of somatic functions. During embryogenesis it is required for asymmetric cell division of neuroblast and muscle precursor stem cells and appears to function by promoting the localized accumulation of atypical Protein Kinase C (aPKC) [19]. In addition, *orb2* mutants have substantially reduced viability, a shortened life span, and defects in behavior and long-term memory [14], [19], [20], [21]. Here we report that *orb2* is essential for spermatogenesis, and that it functions in programming the orderly and sequential progression of spermatogenesis from meiosis through differentiation.

## Results

### Orb2 expression pattern during spermatogenesis

**Figure 1:**
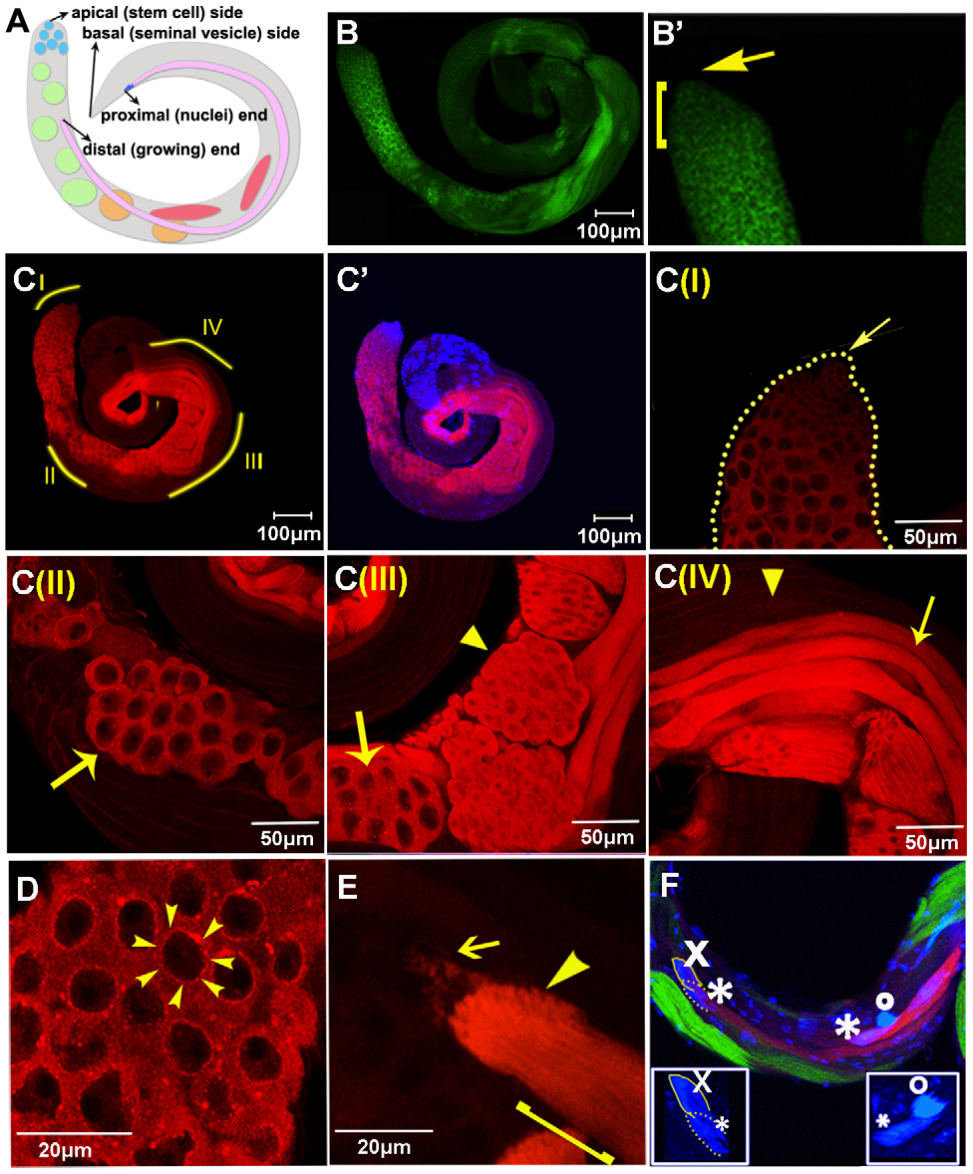
Orb2 expression during spermatogenesis. A) Drawing of the testis. Light blue: stem cell and spermatogonia region, green: spermatocytes, orange: spermatids, red: beginning of spermatids elongation, purple: elongated spermatids (dark blue: spermatid nuclear bundle). Terms used for describing directions of elongation are indicated here. B) Florescent *in situ* hybridization with *orb2* antisense probe of one testis. Image is stitched together from 4 frames with Fiji. B′) Zoom in view of the tip region. Arrow indicates position of the stem cell niche; square bracket outlines the spermatogonia region. C, C′) Testis stained with Orb2 antibody (red) and overlay of Orb2 and DNA (blue). C(I): Testis tip and spermatogonia region containing the 2,4,8-cell cysts. Arrow indicates position of the stem cell niche. C(II): Mature spermatocytes (arrow). C(III): Spermatocytes (arrow) and spermatids (arrowhead). C(IV): elongating spermatids. Arrow marks those with Orb2 expression, and arrowhead marks those without Orb2. D) Orb2 is concentrated around the growing spermatocyte nuclei. Arrowheads mark one nucleus. E) Orb2 localization near the distal (growing) tip of the elongating flagellar axonemes. Arrow, arrowhead and square bracket labels the leading edge of the axonemes, Orb2 concentrated region, and extended Orb2 expressing region respectively. F) Co-labeling of Orb2 in DJ-GFP testes. Orb2 is detected in cysts in which the 64 spermatid nuclei have coalesced into the nuclear bundle (*), but have not yet fully completed chromosome condensation (o). DJ-GFP is only expressed in spermatid cysts that have condensed their chromosomes. Some fully elongated spermatids with fully condensed nuclear bundles have neither Orb2 nor DJ-GFP (x). Insets are enlarged from the main panel. Scale bars are as shown in each panel.

*In situ* hybridization and antibody staining were used to examine *orb2* expression in the testes. While there is little if any *orb2* mRNA (Figure 1B, 1B′) or protein (Figure 1C, 1C(I)) in stem cells, low levels are detected in the mitotic cysts. After mitosis is finished and the interconnected spermatocytes begin to grow, there is a substantial upregulation in both mRNA and protein. This period of growth corresponds to the stage when many gene products needed for subsequent steps in spermatogenesis are synthesized [22], [23]. Though Orb2 protein is found throughout the spermatocyte cytoplasm, higher levels of protein are concentrated in a ring around the nucleus (Figure 1C(II), 1D). Orb2 expression peaks as the mature spermatocytes go through meiosis and high levels of Orb2 are found in 32 and 64 cell spermatid cysts (Figure 1C(III)).

Orb2 persists after the spermatids in the 64 cell cysts start differentiation and begin flagellar axoneme elongation (Figure 1C(IV)). As the axonemes begins to elongate, the 64 spermatid nuclei bundle together and then begin to condense into needle-like structures (Figure 1F, inset, Figure 1A). Though Orb2 is distributed along the entire axoneme bundle, the highest concentrations are found in a prominent band (Figure 1E, arrowhead) close to the distal tip of the growing flagellar axonemes (Figure 1A). The leading edge of the axonemes is just in front of the Orb2 band and this region contains small clumps of Orb2 (Figure 1E, arrow). In the region behind the band, Orb2 is organized into a series of striated lines that extend towards the sperm nuclei at the proximal (basal) tip of the spermatid (Figure 1E, bracket) and presumably correspond to individual flagellar axonemes in the spermatid bundle.

**Figure 2:**
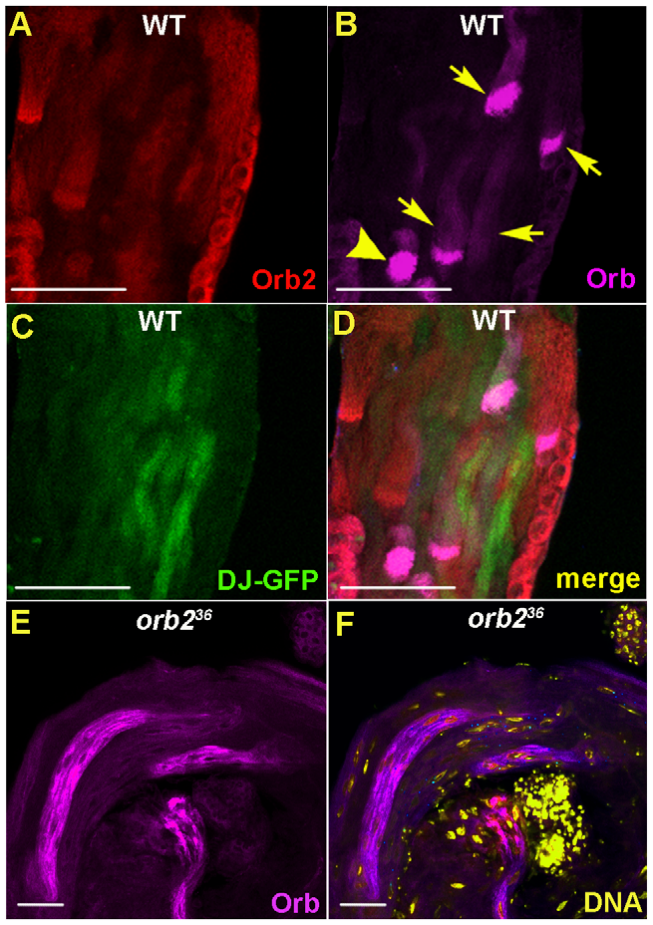
Orb expression in wild-type and *orb2^36^* testes. A–D) Confocal images showing whole mount staining of testes with Orb (pink) and Orb2 (red) antibodies in DJ-GFP (green) expressing wild type testis. Arrow shows that Orb is expressed at the tip of those spermatids that express neither Orb2 nor DJ-GFP. Orb expression is sometimes seen in spermatids that have residual Orb2 (arrowhead). E, F) In *orb2^36^* testes, Orb expression is observed in spermatids that are not fully elongated and its preferential localization near the tip of the elongating flagellar axonemes is lost. Orb: pink; DNA, yellow. Scale bar, 50 µm.

While Orb2 is present in elongating spermatids that have not yet completed nuclear condensation (* in Figure 1F), it disappears once elongation and nuclear condensation are completed (o in Figure 1F). To confirm this, we compared the accumulation patterns of Orb2 and Don Juan-GFP (DJ-GFP). While DJ-GFP is highly expressed once the nuclei have condensed and individualization begins, it is not found in spermatids that are still undergoing elongation [24], [25]. As expected, we did not observe spermatids that simultaneously had Orb2 and DJ-GFP. Moreover, since some fully elongated spermatids with condensed nuclear bundles have neither Orb2 nor DJ-GFP (x in Figure 1F), there seems to be a delay between the disappearance of Orb2 and DJ-GFP expression. This suggestion is supported by a comparison of the Orb2 and Orb expression patterns. *orb* is transcribed post-meiotically and *orb* mRNAs localize in a band at the distal tip of elongating spermatids [3], [26]; however, the localized mRNAs does not appear to be translated until the end of the elongation phase after Orb2 begins to disappear. Figure 2A–2D show that high levels of Orb are found in the tips of elongated spermatids that have neither Orb2 nor DJ-GFP. On occasion we observed spermatids that have activated Orb translation but still retain some residual Orb2 (Figure 2B, arrowhead).

### Orb2 phosphorylation states are changed in *aly* and *can* class mutants

While meiosis and differentiation require different gene products for execution and have their own regulators, there is a class of genes that control both aspects of spermatogenesis. Included in this group are *always early* (*aly*), *spermatocyte arrest* (*sa*), *meiosis I arrest* (*mia*) and *cannonball (can)* which encode testes specific TAFs (TATA Box Protein associated factors) [22], [27]. Mutations in these testes specific TAFs cause spermatocytes to arrest at the G2-M transition of meiosis I and block the expression of factors needed for differentiation [28]. However, though these genes encode factors essential for Pol II activity, the effects of mutations are not limited to general transcription. For example, *twine* (*twe*) mRNA is expressed in tTAF mutants, but is not properly translated [28]. Figure S1 shows that mutations in these four genes have two effects on Orb2 protein expression in the testes. First, Orb2 levels were substantially reduced (Figure S1A). Second, there was a noticeable reduction in the electrophoretic mobility of the larger Orb2 isoform. As illustrated for the *sa* mutation, treatment of the testes extract with lambda phosphatase removes the Orb2 signal with slow electrophoretic mobility and indicates that phosphorylation is responsible for the reduced mobility of Orb2 in the mutant testes (Figure S1B). As might be expected, these tTAF mutations do not seem to affect Orb2 in somatic tissues such as the head (Figure S1A).

### *orb2* mutants are male sterile

**Figure 3:**
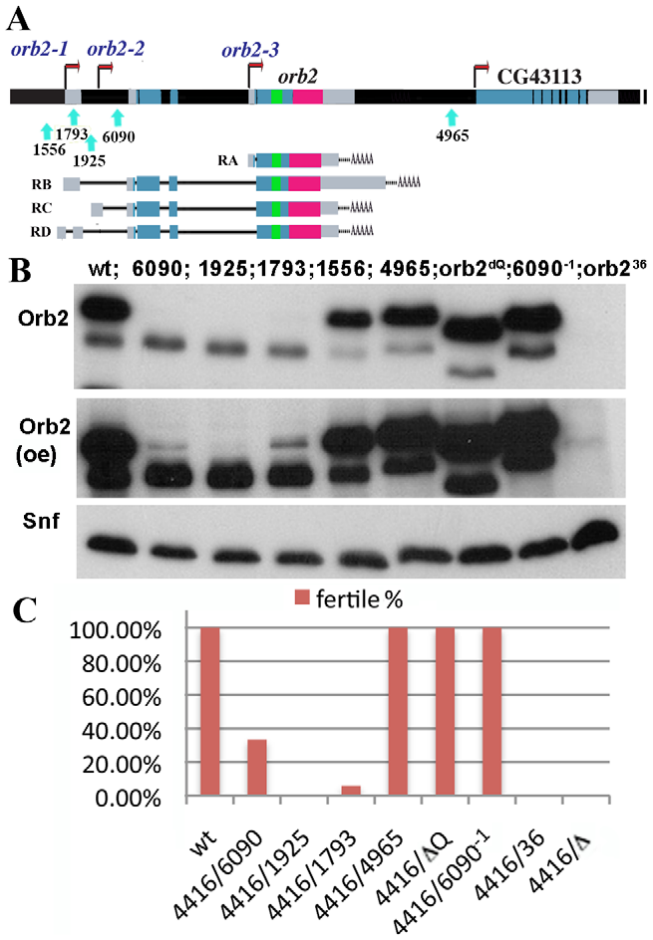
Orb2 expression in the testes and fertility of different *orb2* alleles. A) *orb2* gene structure. *orb2* has three promoters (*orb2-1*, *2*, and *3*) and encodes multiple transcripts. *piggyBac* insertions *1556*, *1793*, *1925*, 6090, and *4965* are marked by green arrows. Only *1793*, *1925*, and *6090* are expected to disrupt *orb2* transcripts. Green box marks the poly-Q sequence that is deleted in *orb2^ΔQ^* allele [14]. See Figure S1 and [19] for further details. B) Orb2 expression in *piggyBac* alleles (6090, 1925, 1793, 1556, 4965), *orb2^ΔQ^*, *piggyBac* insertion revertant (*6090^−1^*), and *orb2^36^*. (oe): over exposed. C) Fertility of *piggyBac* insertion alleles, revertant, *orb2^ΔQ^*, *orb2^36^ and orb2^Δ^*. Fertility is consistent with Orb2 expression in the testes. *DF(3L)4416* (referred to as 4416) is a small deletion allele that removes part of the third chromosome that includes *orb2* gene region.

To better understand how *orb2* functions in spermatogenesis, we examined the effects of mutations. Previously we characterized a collection of 5 transposon insertions in the *orb2* locus [19]. As shown in Figure 3, two of the transposons, *1556* and *4965* have no effect on Orb2 expression. This is expected as *1556* is inserted upstream of the *orb2*-*1* promoter, while *4965* is located downstream of the Orb2 protein coding sequences. Two of the transposons, *6090* and *1925*, are inserted downstream of both the *orb2-1* and *orb2-2* promoters and interfere with expression of *orb2* mRNAs encoding the 75 kD isoform in the testes and head (Figure 3, Figure S2 and [19]). In contrast, the *1793* insertion, which is located farther upstream in between the *orb2-1* and *orb2-2* promoters, affects 75 kD expression in the testes, but not in the head, suggesting that *orb2-1* is more heavily used in the testes, while *orb2-2* is more heavily used in the head (Figure S2). As expected from their insertion sites, none of the transposons affect the 60 kD isoform. On the other hand, the reduction in the 75 kD isoform in *6090*, *1925* and *1793* is accompanied by a small but reproducible increase in the 60 kD isoform (Figure 3B). This raises the possibility that a negative feedback loop might regulate the levels of the two isoforms.

Consistent with an important role for Orb2 in spermatogenesis, we find that the fertility of homozygous *6090*, *1925*, and *1793* males is substantially impaired (not shown). When *trans* to deficiencies that uncover *orb2*, *1925* is completely sterile, while *6090* and*1793* occasionally give fertile males (Figure 3C). In contrast, the two insertions, *1556* and *4965*, that have no effect on the expression of the 75 kD isoform, are fully fertile. That sterility is due specifically to the loss of the Orb2 75 kD isoform is supported by the finding that excision of the transposon insertions restores the expression of this isoform and reverts the sterility phenotype (*6090^−1^*, Figure 3).

Since the mutants still expressed the 60 kD isoform, along with residual 75 kD isoform, they could retain some *orb2* function. For this reason, we generated *orb2* nulls using FLP recombination (Figure S2) [29], [30]. Two upstream *piggyBac* insertions (*1556* and *1925*) contain correctly oriented FRT sites for deleting the *orb2* protein coding sequence when paired with the downstream *4965* insertion. The resulting deletions, *orb2^7^* (*1925×4965*) and *orb2^36^* (*1556×4965*), eliminate *orb2* mRNA and protein expression (Figure 3). They have substantially reduced viability (data not shown), while the surviving males are completely sterile (Figure 3). To exclude possible background effects, we combined the two null alleles with three different third chromosome deficiencies that remove small parts of the third chromosome including *orb2* (*Df(3L)ED4421*, *Df(3L)ED4415*, and *Df(3L)ED4416*). These *trans* combinations also have reduced viability and are completely male sterile (Figure 3C; not shown). Similar results were obtained for an independently generated null allele, *orb2* **^Δ^** [14]. Since all null alleles behave the same in our assays, we used *orb2^36^* in the experiments described below.

### *orb2* mutant *s*permatocytes fail to complete meiosis

**Figure 4:**
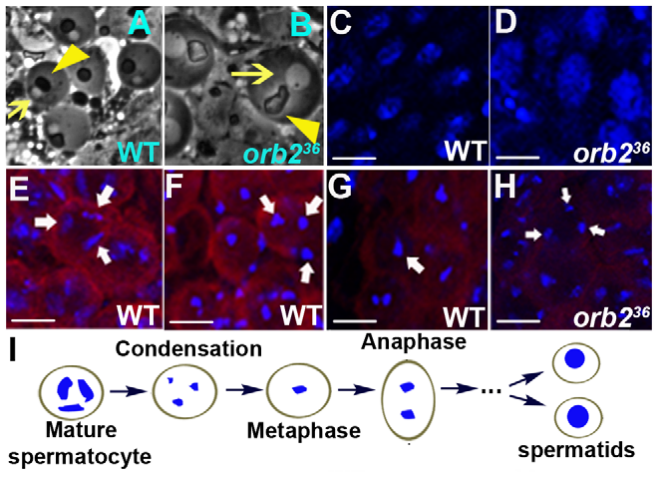
*orb2^36^* spermatocytes fail to undergo the G2-M transition of meiosis I. A, B) Wild type and *orb2^36^* spermatids. *orb2^36^* spermatids are larger than wild type, have a much larger nucleus (arrow) and an irregularly shaped Nebenkern (arrowhead). C, D) Wild type and *orb2^36^* spermatids stained with Hoechst to visualize nuclear size and DNA content. E, F, G) The process of chromosome condensation in wild type spermatocytes in G2 of meiosis I. E) Mature spermatocyte stage. F) Condensation phase. G) Onset of metaphase I. H) Only partially condensed chromosomes corresponding to the wild type panel F are observed in *orb2^36^*. I) Diagram of chromosome condensation during meiosis I. Scale bar: 10 µm.

Overall testes morphology and the pre-meiotic stages of spermatogenesis appear normal in *orb2^36^* and other *orb2* mutants. The spermatogonia undergo the sequential mitotic divisions generating 16 interconnected spermatocytes, and the spermatocytes mature as in wild type. However, subsequent stages of spermatogenesis are abnormal. In wild type, the products of meiosis, the spermatids in the 64 cell cysts, have two characteristic spherical structures when observed by phase contrast microscopy: a light nucleus and a dark mitochondrial Nebenkern (Figure 4A). While pseudo-spermatids are present in *orb2^36^*, the cells and their nuclei are unusually large and they have a poorly contrasted Neberken, which is abnormally shaped and sometimes fragmented (Figure 4B). As the overall DNA content is also increased (Figure 4C, 4D), it seems likely that the *orb2* spermatids have replicated their DNA as in wild type, but failed to complete meiotic divisions. Consistent with this possibility, we never observe products of the first and second meiotic divisions, the 32 and 64 cell cysts respectively, in *orb2^36^* testes. By contrast, 32 and 64 cell cysts are seen in wild type.

To further characterize the meiotic defects, we examined chromosome morphology. During the prolonged G2 before the spermatocytes enter meiosis I, the three large chromosomes segregate into 3 domains and start the process of condensation. As illustrated in Figure 4I, the spermatocyte chromosomes initially coalesce into irregular rod-like structures located at vertices of a triangle (Figure 4E). They subsequently condense into 3 sharp dots (Figure 4F) before congressing to the metaphase plate in preparation for the first meiotic division (Figure 4G) [31]. In *orb2*, the spermatocyte chromosomes segregate into three domains, and start the process of condensation. However, condensation is incomplete and the chromosomes don't congress to the metaphase plate (Figure 4H).

### Nuclear Cyclin A accumulates in *orb2* spermatocyte cysts

**Figure 5:**
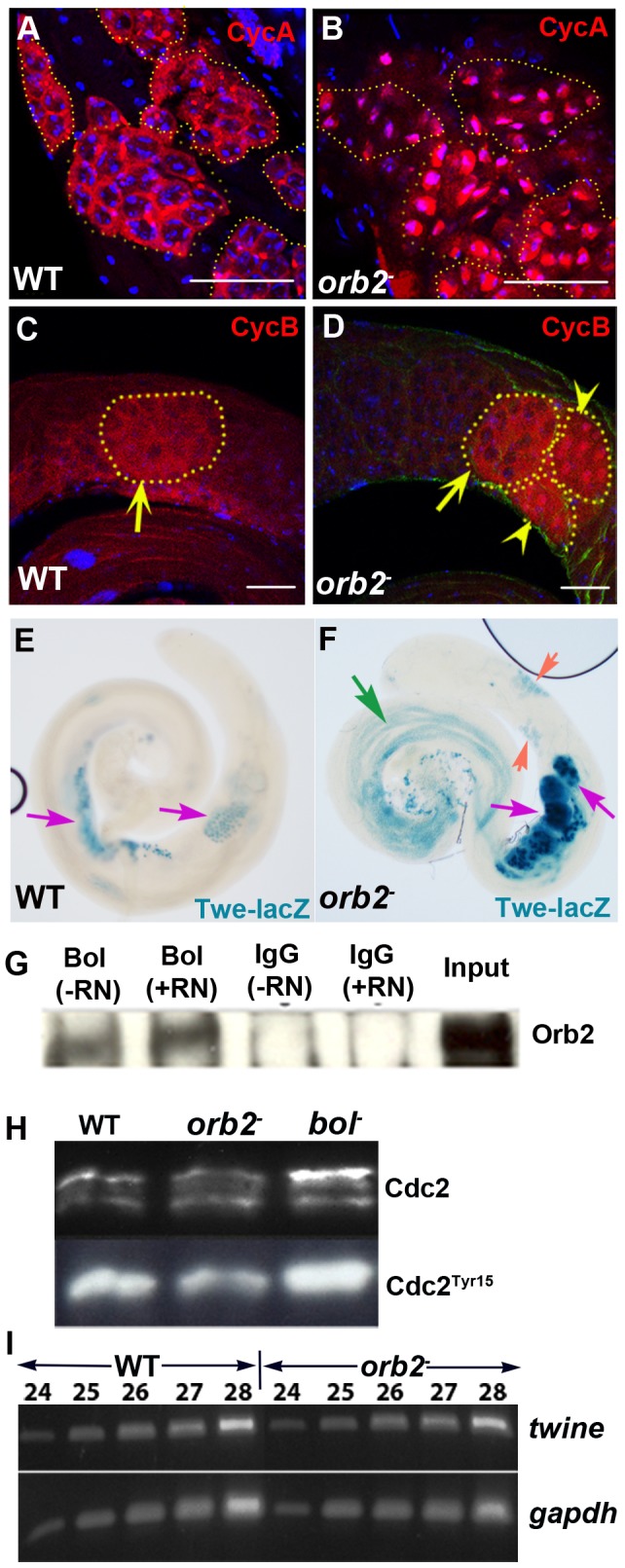
*orb2^36^* cysts arrest meiosis I at a step after the expression of the Twine phosphatase. A) Spermatocyte cysts in wild type typically have cytoplasmic CycA. B) *orb2^36^* testes have multiple spermatocyte cysts with nuclear CycA, as observed in *bol* [34]. C) In wild type spermatocyte cysts, CycB is typically only found in the cytoplasm. D) CycB shows a similar nuclear enrichment as CycA in *orb2^36^*. Arrow marks wild type and *orb2^36^* cysts with cytoplasmic CycB, while arrowhead points to two *orb2^36^* cysts with nuclear CycB. In contrast to *orb2*, cysts with cytoplasmic, but not nuclear CycB were seen in *bol* mutants (Xu, unpublished results). E, F) Twe-lacZ is over-expressed in *orb2^36^*. Purple arrows: *orb2^36^* cysts have much higher levels of Twe-lacZ than wild type. Orange arrow: *orb2^36^* cysts containing immature spermatocytes prematurely express Twe-lacZ. Green arrow: Twe-lacZ persists in elongating *orb2^36^* spermatids. G) Orb2 and Bol are found in the same complex in testes extracts. Testes extracts were immunoprecipitated with Bol or control IgG, in the presence or absence of RNase (RN) as indicated, and Westerns of the immunoprecipitated proteins were then probed with Orb2 antibodies. H) Testes extract from wild type (WT), *orb2* and *bol* were probed with antibodies against bulk Cdc2 (top) and phospho-Tyr15 Cdc2 (bottom). Note that *bol* and *orb2* have opposite effects on CDC2 Tyr15-P. The levels of phosphorylated Cdc2 in *bol* testes are higher than wild type, whereas they are lower than wild type in *orb2*. Ratio of phosphorylated Tyr15-P to unphosphorylated is as follows: WT = 1.5; *orb2* = 0.8; *bol* = 2.5. I) Semi-quantitative RT-PCR of *twine* and *gapdh* mRNA showing that *twine* mRNA levels in *orb2^36^* are equivalent to wild type. Scale bar: 50 µm.

These findings suggest that *orb2* spermatocytes arrest meiosis at a step prior to the first meiotic division. To analyze the meiotic arrest further we examined Cyclin A accumulation. In wild type testes, Cyclin A accumulates in the cytoplasm during G2. However, just prior to the meiosis I G2 to M transition, Cyclin A is targeted to the spermatocyte nucleus, and then quickly degraded as meiosis proceeds [31], [32]. Since nuclear localization is only transient, cysts with nuclear Cyclin A are rarely seen in wild type (Figure 5A). However, in *orb2^36^* and *orb2^36^/Df(3L)4416*, most cysts in the middle of the testes have high levels of nuclear Cyclin A (Figure 5B).

### Orb2 is in a complex with the translational regulator Boule

These *orb2* meiotic phenotypes are similar to the phenotypes reported for mutations in *boule* (*bol*) and *off-schedule* (*ofs*) [32]–[34]. *bol* encodes a homolog of mammalian DAZ fertility factor, while *ofs* encodes a testes eIF4G. Like *orb2*, *bol* and *ofs* mutant spermatocytes arrest meiosis prior to the first meiotic division and the cysts have high levels of nuclear Cyclin A. The fact that all three proteins are needed for meiosis suggested that they might function together. To explore this possibility, we first tested whether Orb2 and Bol associate with each other in testes extracts. As shown in Figure 5G, Orb2 and Bol are in an RNase resistant immunoprecipitable complex.

We also examined the pattern of Bol accumulation in *orb2^36^* testes. In wild type spermatocytes, Bol localizes in a perinucleolar dot during spermatocyte maturation; however, once meiosis begins, Bol is relocalized to the cytoplasm where it is thought to promote the translation of target mRNAs [35]. Figure S3A, S3A′, S3B, and S3B′ show that both phases of Bol localization are observed in *orb2* mutant testes. Also as in wild type, Bol is present in “post-meiotic” (see below) *orb2^36^* spermatids even though they haven't undergone meiosis (Figure S3C, S3C′, S3D and S3D′). As for Ofs, we were unable to demonstrate an association with Orb2 in testes extracts (Xu: unpublished data).

### *twine* is misexpressed in *orb2* mutant testes

One reason that *bol* and *ofs* mutants are blocked in meiosis at the G2/M transition is that both factors are required for translation of *twine* (*twe*) mRNA [33], [34], [36]. *twe* encodes *Drosophila* Cdc25 phosphatase. In order for meiosis to proceed *twe* must remove an inhibitory phosphorylation on tyrosine 15 of Cdc2 (Ck1) [37], [38]. In *bol* testes, *twe* mRNA is present but it is not translated. In the absence of Twe protein, phosphorylated Cdc2 on Tyr15 accumulates and meiosis arrests at the G2/M transition [38]. Since our results indicate that *orb2* also arrests meiosis at the G2/M transition, we anticipated that *orb2* activity would be required to translate *twe* mRNA. To test this hypothesis, we first determined whether *twe* mRNA levels are normal. The RT-PCR experiment in Figure 5I shows that *twe* mRNA levels in *orb2* testes are similar to wild type. We next used a chimeric *twe*-lacZ translational reporter to ascertain whether *twe* mRNA is translated in *orb2* mutants. The reporter has sequences encoding *β-galactosidase* inserted in frame into the *twe* gene and expresses a chimeric mRNA including the *twe* 3′ UTR [36]. While we anticipated that the translation of the chimeric *twe-lacZ* mRNA would be blocked in *orb2* testes as in *bol* (and *ofs*), this is not the case. Instead, Twe-lacZ expression in *orb2* exceeds even wild type.

Figure 5E and 5F show that the pattern of Twe-lacZ expression differs in several respects from wild type. First, compared to wild type (E) there are many more cysts in *orb2* testes (F) that express Twe-lacZ. Second, the amount of lacZ is typically much higher than in wild type (compare purple arrows in E and F). Third, while residual Twe-lacZ is degraded in wild type once meiosis is complete and the spermatids begin differentiation, it persists in elongating *orb^36^* spermatids (green arrow in Figure 5F). Finally, we sometimes observe that Twe-lacZ is precociously expressed in immature spermatocytes that normally would not have Twe protein (orange arrows in Figure 5F).

Meiosis arrests at the G2-M transition in *bol* mutants because CDC2 remains phosphorylated on Tyr15 in the absence of Twe [37], [38]. This should not be the case in *orb2* because high levels of Twe-lacZ and presumably Twe accumulate. To confirm this prediction we compared CDC2 Tyr15-P in wild type, *bol* and *orb2* testes. As expected the ratio of phosphorylated to unphosphorylated CDC2 is elevated in *bol* mutants compared to wild type, while it is reduced in *orb2* (Figure 5H). This finding indicates that CDC2 is activated in *orb2* mutants and that meiosis I must be blocked at a subsequent step in the G2-M transition.

### Nuclear Cyclin B accumulation in *orb2* testes

To further pinpoint the meiosis block we examined the expression of Cyclin B (Cyclin B). In wild type testes Cyclin B is expressed in primary spermatocytes when chromosome condensation starts. It persists during metaphase and is abruptly degraded at the beginning of anaphase [28]. Like Cyclin A, Cyclin B's transient nuclear accumulation is seen only very infrequently. Previous studies have shown that the upregulation of Cyclin B expression during chromosome condensation doesn't occur in *ofs* mutants. But other than that, Cyclin B expression and degradation seem normal [33], [34]. In *bol* testes, Cyclin B is found in the cytoplasm (Xu, unpublished data). In *orb2* mutant testes, Cyclin B initially accumulates in the cytoplasm as in wild type (Figure 5C, 5D, arrow). However, instead of transiently accumulating in the nuclei and then disappearing, we find many *orb2* cysts with high levels of nuclear Cyclin B (Figure 5D, arrowhead). In older *orb2* cysts we often observe many small Cyclin B speckles in the cytoplasm. Taken together with the effects on *twe* expression and CDC2 phosphorylation, these findings place the meiosis arrest in *orb2* at a step later than in *bol* and *ofs*.

### Sperm differentiation is disrupted in *orb2* mutants

**Figure 6:**
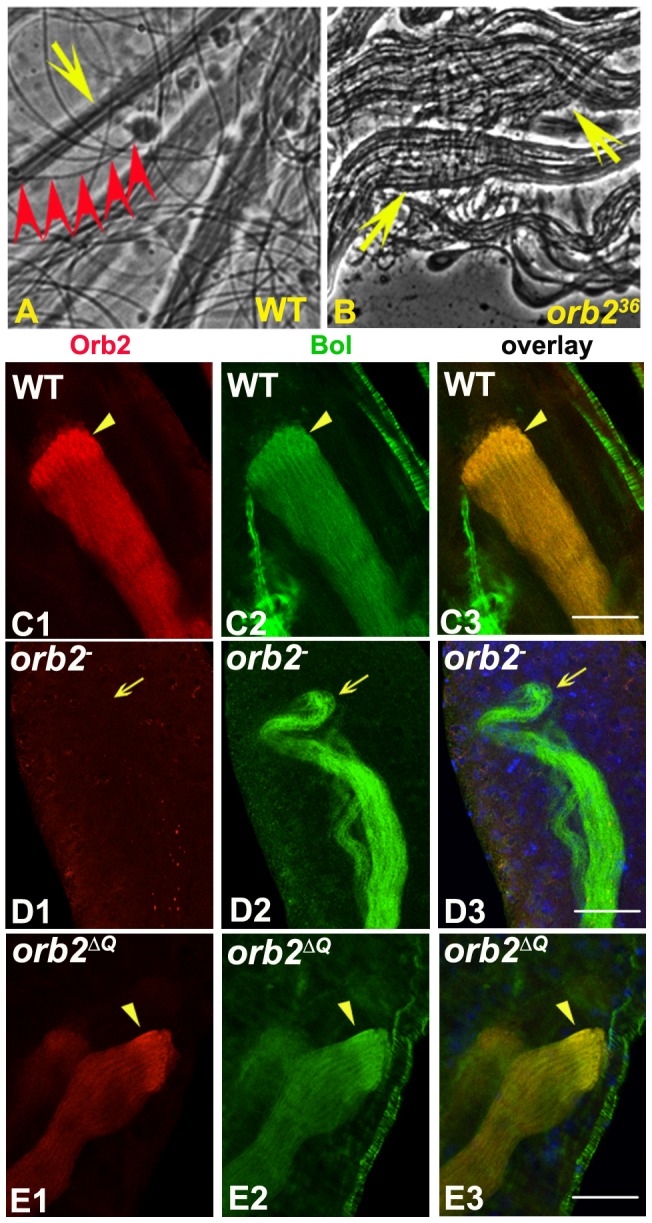
*orb2^36^* spermatids have defects in flagellar axoneme elongation. A, B) Phase contrast images showing wild type (A) and *orb2^36^* (B) elongated spermatid bundles. Wild type spermatid flagellar axoneme bundles (arrow) have a smooth morphology and extend in a nearly straight line. *orb2^36^* bundles have rough and uneven morphology, are shorter, and zigzag back and forth. Individualized sperm (red arrowheads in A) are observed in wild type but not *orb2^36^* testes. C1–C3) Wild type testes double stained with Orb2 (red) and Bol (green) antibodies showing co-localized Bol and Orb2 concentrated in a band near the tip of the elongating flagellar axonemes (arrowhead) and decreased expression level following this band. D1–D3) Bol localization at the tip is lost in *orb2^36^* flagellar axonemes (arrow), while there is an uneven distribution along the flagellar axonemes extending behind the tip. E1–E3) Orb2 and Bol co-localization at the tip of the elongating flagellar axonemes in *orb2^ΔQ^* is as in wild type. Scale bar: 20 µm.

Even though *orb2* spermatocytes fail to undergo meiosis, the spermatids in the older cysts eventually exit the meiotic cycle and begin the process of differentiation. One of the first steps in differentiation is the elongation of the flagellar axonemes. In wild type, the elongating bundle of flagellar axonemes extends towards the apical tip in a roughly straight and smooth line (Figure 6A). In contrast, the elongating flagellar axonemes in *orb2* zigzag back and forth and are much shorter than wild type. The individual axonemes also often splay out from each other instead of remaining in a tight bundle (Figure 6B). In addition, rather than having a smooth, regular internal morphology, their internal morphology is rough and irregular. This phenotype likely arises from underlying defects in the assembly or localization of axonemal proteins. One protein that is not properly localized is the meiosis regulator Bol. In wild type, Bol co-localizes with the prominent Orb2 band near the tip of the elongating flagellar axoneme bundle. In the region distal to this band extending towards the spermatid nuclei, there is a lower level of Bol and Orb2 and both are distributed uniformly along the individual axonemes (Figure 6C1–6C3). In *orb2* testes, the prominent Bol band at the tip of the axoneme is missing, while in the remainder of the axoneme bundle, Bol is dispersed in an irregular fashion, and unlike wild type, its association with individual axonemes is difficult to discern (Figure 6D1–6D3).

**Figure 7:**
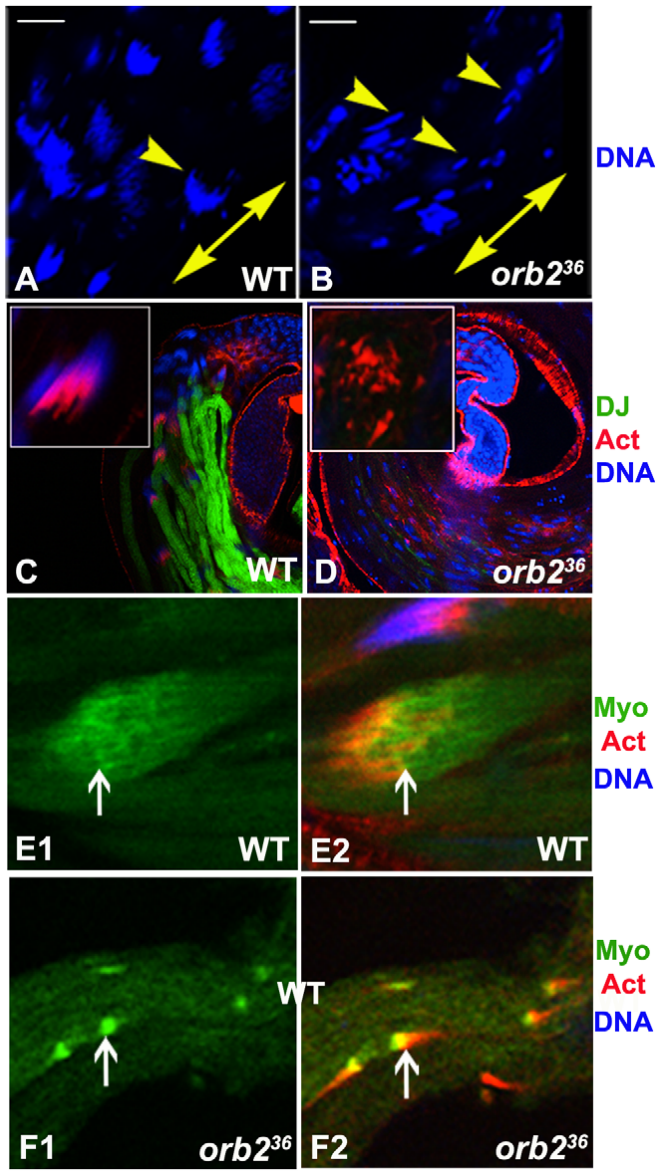
Spermatid nuclear bundles and Individualization Complex are not properly assembled in *orb2^36^*. A, B) Formation and compaction of spermatid nuclear bundles in wild type (WT) and *orb2^36^*. Arrowhead in A labels a condensed spermatid nuclear bundle. Arrowheads in B mark incompletely assembled spermatid nuclear bundles in *orb2^36^*. Note that individual needle-shaped spermatid nuclei are larger than wild type, and most of the nuclei are scattered along the partially elongated spermatids. Double arrow indicates direction of spermatid elongation. C, D) Individualization complexes and DJ-GFP are missing from *orb2^36^* testes. IC (Actin): red; DJ-GFP: green; DNA, blue. Inset shows complete IC (C) or scattered actin cones (D) in either wild type or *orb2^36^* testes. E1, E2, F1, F2) MyosinVI (green) is a component of the Actin cones and is localized before the triangle shaped Actin signal in wild type IC (E1, E2, arrow). MyosinVI is also observed in the scattered actin cones in *orb2^36^* testes (F1, F2, arrow). Scale bar in A and B: 20 µm.

At the end of meiosis just as spermatid elongation commences, the 64 spermatid nuclei cluster together and begin the process of condensation, eventually forming a cap-like structure (Figure 7A, arrowhead) [39]. This doesn't happen in *orb2^36^*, and instead of coalescing into a tight bundle, the spermatid nuclei usually end up spread out along the partially elongated flagella axonemes (Figure 7B). The process of individualization begins once elongation is complete. In wild type testes, individualization is accomplished by a special structure called the Individualization Complex (IC). The IC is comprised of 64 individual actin cones that assemble around each nucleus in the condensed spermatid nuclear bundle (Figure 7C, inset) and then travels down the bundled axonemes, ensheathing each in a plasma membrane and pushing the excess cytoplasm into a waste bag [40], [41]. The IC is never assembled in *orb2^36^* testes and individualization never takes place. However, we do observe scattered triangular shaped actin cones (Figure 7D, inset). Based on the observed defects, the steps involved in organizing actin filaments into individual actin cones might be comparatively normal, while the subsequent assembly of the cones into the larger IC ensemble is not. Consistent with this idea, Myosin VI, a component of the Actin cone [39], is present in the *orb2^36^* cones (Figure 7E1, 7E2, 7F1, 7F2). As the bundled and condensed spermatid nuclei are believed to provide the scaffolding for assembling the IC [41], the defects in *orb2^36^* could be due to the failure in spermatid nuclei bundling and condensation. Alternatively or in addition, *orb2* may be regulating genes directly involved in assembling the IC. As might be expected from the failure in IC assembly, Don-Juan GFP is not expressed in *orb2^36^* testes (Figure 7C, 7D) and mature sperm are never observed (Figure 6A, 6B).

### Orb2 represses Orb expression

**Figure 8:**
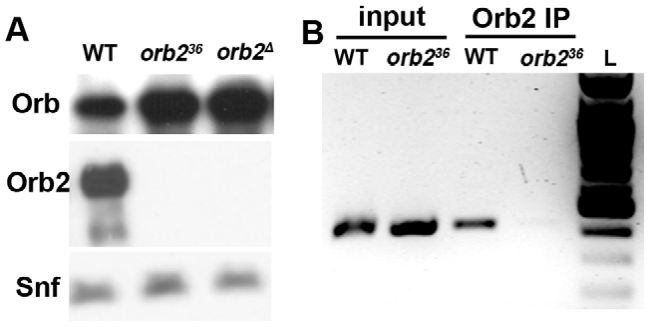
*orb* is an Orb2 regulatory target. A) Western blots of extracts prepared from wild type, *orb2^36^* and *orb2^Δ^* testes were probed as indicated on the left. In this experiment Snf (Sans filles) was used as a loading control. Similar results were obtained using Tubulin as the loading control. B) *orb* mRNA can be immunoprecipitated with Orb2 antibodies from wild type testes extracts. *orb2^36^* testis extract are used as a negative control for immunoprecipitation. After reverse transcription using oligo-dT primers, the *orb* cDNA was amplified using a primer set from the 3′ end of the male *orb* mRNA. L: 100 bp DNA ladder.

*orb* mRNAs are expressed after meiosis is complete and localize to the tip of the elongating axoneme close to the band of Orb2 protein [3], [26]; however, these localized mRNAs don't appear to be translated until Orb2 protein begins to disappear at the end of the elongation phase (Figure 2A–2D). These observations suggested that the localized *orb* mRNAs might be a target of Orb2 repression. To test this hypothesis, we first probed Western blots of wild type and *orb2* testes extracts with Orb antibodies. Figure 8A shows that Orb levels are elevated in *orb2* mutants. In addition to this increase in Orb protein, *orb* mRNA translation appears to be ‘prematurely’ activated in *orb2* mutant spermatids. As shown in Figure 2E and 2F, Orb protein is expressed in incompletely elongated *orb2* mutant spermatids. Finally, the expression of Orb protein is not properly restricted to the tip of the elongated flagellar axoneme as in wild type. Instead, Orb is found throughout the mutant spermatid axonemes.

The *orb* mRNAs in the two sexes differ at their 5′ and 3′ ends. The male transcripts begin at an internal promoter and encode a protein that has a different N-terminus from the female Orb. At the 3′ end, the male UTR is only about 200 bases in length, while the female UTR is over a thousand [3]. While the male 3′UTR lacks most of the critical sequences for *orb* mRNA localization and translational regulation in ovaries, there are two CPE elements. Thus, it seemed possible that *orb2* might repress *orb* mRNA translation by a mechanism that involves an association between Orb2 protein and *orb* mRNA. To test this idea we reverse transcribed RNA isolated from Orb2 immunoprecipitates of wild type and *orb2^36^* testes extracts, and then used primers specific for the *orb* male 3′ UTR for PCR amplification. Figure 8B shows that *orb* mRNA is readily detected in the Orb2 immunoprecipitates from wild type but not *orb2^36^* testes. In control experiments (not shown), neither *boule* nor *twine* mRNA was found in Orb2 immunoprecipitates. Taken together, these findings are consistent with the idea that Orb2 represses *orb* mRNA translation directly, rather than by regulating some other intermediate.

More than twenty other mRNAs are transcribed post-meiotically and localize to the tip of the elongating spermatid flagellar axonemes [26]. In addition to having similar expression and localization patterns to *orb* several of these mRNAs have CPE-like elements in their 3′ UTRs and could be regulatory targets of *orb2*. Consistent with this possibility we found that two of the CPE containing mRNAs, *scotti* and *f-cup*, can be immunoprecipitated with Orb2 antibody from wild type but not *orb2* mutant testes (Figure S4). While the function of *f-cup* is unknown, Barreau *et al.* [26] found that *scotti* mutant males are sterile. The primary defect appears to be at a late step in spermatogenesis and involves the assembly or maintenance of the IC structure.

### Uncoupling *orb2* functions in meiosis and differentiation

Although the experiments above show that *orb2* is required for spermatid differentiation, it could be argued that the differentiation defects are the indirect consequence of the failure to undergo meiosis rather than because *orb2* has special functions in this stage of spermatogenesis. To address this problem, at least in part, we took advantage of the hypomorphic *orb2^ΔQ^* allele, which has a small deletion that removes an N-terminal poly-Q domain (Figure S2A, Figure 3) [14]. As shown in Figure 3B, both the large isoform and the smaller, testes specific isoform are abundantly expressed in *orb2^ΔQ^* testes; however, they migrate more rapidly than the corresponding wild type isoforms due to the loss of the poly-Q domain.

**Figure 9:**
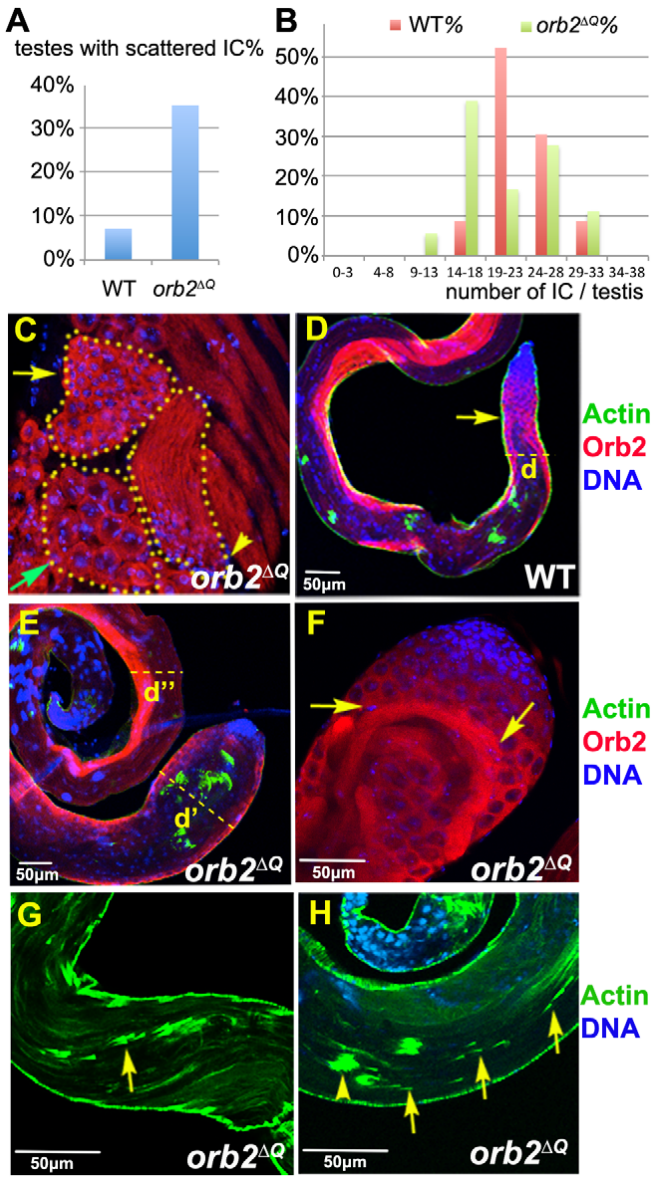
Orb2 functions in meiosis and differentiation can be uncoupled in *orb2^ΔQ^* allele. A) Percentage of scattered ICs is higher in *orb2^ΔQ^* than wild type. A total of 34 wild type and 46 *orb2^ΔQ^* testes were counted. B) Percentage of wild type or *orb2^ΔQ^* testes having 0–3, 4–8, 9–13, 14–18, 19–23, 24–28, 29–33 or 34–38 ICs per testes. *orb2^ΔQ^* testes have fewer ICs compared to wild type. C) *orb2^ΔQ^* testes have normal spermatids. Green arrow: mature spermatocytes; yellow arrow: 64-cell spermatids cyst; arrowhead: spermatids at the beginning of elongation. D) Wild type testes stained with Orb2 (red), IC (Phalloidin, green) and DNA (blue). Yellow arrow points to where elongation usually stops in wild type testes. “d” marks the normal diameter of a testis at spermatogonia and early spermatocytes region. E–F) Flagellar axoneme bundles in *orb2^ΔQ^* testes are over elongated. E) Overgrowth results in the swelling of the testis tip. Diameter of the *orb2^ΔQ^* spermatogonia part of the testis is larger than that of the wild type (compare d in D and d′ in E), while the diameter of the nuclei side is relatively normal (compare d in D and d′, d″ in E). F) Another example of overly elongated flagellar axoneme bundles in *orb2^ΔQ^* testis tip. The Orb2 positive axoneme bundle extended to the spermatogonial region and then changed its direction of elongation (arrows) to continue growing in the wrong direction. G, H) IC is not properly assembled in *orb2^ΔQ^*. Phalloidin labeled Actin: green; DNA: blue. Arrow in G points to scattered actin cones of an *orb2^ΔQ^* IC that remain relatively close together in one elongating spermatid cyst. Arrows in H are examples of widely scattered actin cones. In *orb2^ΔQ^* testes with scattered IC, we can also observe what appear to be normal looking ICs, as indicated here by arrowhead in H. Scale bar: 50 µm.

We examined spermatogenesis in *orb2^ΔQ^* homozygous flies. In contrast to the mutants that reduce or eliminate expression of the 75 kD isoform, there are no meiosis defects in *orb2^ΔQ^*. Instead, like wild type, 32- and 64-cell cysts are observed, and each of the spermatids in the 64-cell cysts has a normal looking nucleus (Figure 9C) and Nebenkern (not shown). Also unlike *orb2^36^*, elongating *orb2^ΔQ^* flagellar axonemes have a seemingly normal morphology and as in wild type the mutant Orb2 protein and Bol accumulate together in a prominent band near the growing tip of the flagellar axoneme (Figure 6E1–6E3). Likewise the assembly of the spermatid nuclei into a bundle and their condensation appear to be normal (not shown).

On the other hand, the process of differentiation is not normal in *orb2^ΔQ^*. In wild type testes, elongation of the flagellar axoneme stops before the tail reaches spermatogonia region (Figure 9D, arrow points to end of elongation). This is not true in *orb2^ΔQ^*. About 70% of the mutant testes have over-elongated flagellar axonemes that extend into the spermatogonia region. Elongation doesn't seem to arrest even at this point. Overgrowth of spermatid axoneme results in the swelling of testes tip region and an over-sized testes tip is often observed in *orb2^ΔQ^* testes (Figure 9E, compare d in Figure 9E and d′ in Figure 9D). In some cases, the elongating flagellar axonemes push against the testes wall and cause the muscle layer encasing the apical tip of the testes to rupture (not shown). On other occasions, when the flagellar axoneme bundle reaches the spermatogonia region, it changes direction and begins elongating towards the side of the testes or even reverses direction and elongates towards the base of the testes (Figure 9F).

Another differentiation defect is in the assembly and functioning of the IC. While fully elongated cysts with scattered actin cones are occasionally observed in wild type testes (∼6%), 35% of the fully elongated cysts in *orb2^ΔQ^* testes have scattered actin cones (Figure 9A, 9G, 9H). The IC defects range from actin cones that are not fully coalesced into the IC structure (Figure 9G, arrow) to completely dispersed actin cones (Figure 9H, arrows). These IC phenotypes resemble the phenotypes reported for *scotti* [26]. In the testes that have IC defects, there is always a mixture of both wild type and defective ICs (Figure 9H arrow and arrowhead), which may explain why *orb2^ΔQ^* males are still fertile. Also by comparison, all ICs in *orb2^36^* testes are defective. There is also a reduction in the number of ICs in *orb2^ΔQ^* testes. In wild type flies, over 90% of the testes have more than 19 ICs. In contrast, *orb2^ΔQ^* testes, 44% testes have less than 19 IC (Figure 9B). The fact that meiosis is normal in *orb2* ^ΔQ^, but there are clear defects in both spermatid elongation and individualization, would provide further support for the idea that *orb2* activity is required not only for meiosis, but also for proper differentiation.

## Discussion

Although CPEB family proteins play critical roles in germline development in many species, their germline functions differ between proteins within an organism and also between proteins in different organisms. For example, in *C. elegans*, Fog-1 and the Orb2-like CPB-1 function in the male germline and are required for sex determination and meiosis respectively. A third, Orb-like CPEB, CPB-3 is required for meiosis in females [42]–[44]. Similar functional specializations are evident for *orb* and *orb2*. While *orb* is essential for oogenesis, it is not absolutely required for spermatogenesis as *orb* mutant males produce functional sperm and their fertility is reduced but not eliminated. The opposite sex specificity is exhibited by *orb2*. Though genetic interaction studies (suppression of *orb* haploinsufficiency in the *gurken* dorsal-ventral polarity pathway: see for example [17], [55]) suggest that *orb2* may negatively regulate *orb* in the ovary, *orb2* females are fertile and oogenesis appear to be comparatively normal (Nathaniel Hafer, PhD thesis). In contrast, *orb2* plays an essential role in the male germline, and is required for programming the orderly progression of spermatogenesis from meiosis through differentiation.

How CPEB proteins regulate meiotic progression is best understood in *Xenopus* oocytes. During oocyte maturation, CPEB1 acts as a repressor, blocking translation of mRNAs containing CPE motifs. However, after progesterone stimulation, CPEB1 is converted into an activator by Aurora kinase phosphorylation, initiating translation by stimulating the Gld-2 dependent polyadenylation of target mRNAs. Amongst the targets are mRNAs encoding Mos and the Cyclins B2 and B5. These cyclins activate Maturation Promoting Factor (MPF) which mediates entry into metaphase I. Although CPEB1 is degraded during metaphase I, it induces expression of CPEB4, which is a member of the second CPEB family. CPEB4 subsequently controls the transition to metaphase II by regulating Cyclins B1 and B4 expression [45]–[49]. Interestingly, though mouse CPEB1 is also essential for meiosis in both sexes, it controls meiosis at an earlier step by regulating mRNAs encoding synaptonemal complex proteins [50].

Since there is no recombination in *Drosophila* males, the function(s) of *orb2* in meiosis are necessarily different from those of mouse CPEB1 [48]. Additionally, its role is distinct from that of *Xenopus* CPEB1. While *Xenopus* CPEB1 promotes meiotic progression by activating translation of Cyclin B mRNAs, *orb2* pre-meiotic cysts accumulate high levels of Cyclin B. *orb2* also differs from the fly translation factors *ofs* and *bol*. The meiotic phenotypes of mutations in these two genes suggest that they regulate different targets and likely function at earlier steps in meiotic progression than *orb2*. Unlike *orb2* mutants, Cyclin B levels aren't properly upregulated during G2 in *ofs* mutants. However, it is not clear whether *ofs* regulates *Cyclin B* mRNA translation directly, or whether the defects are an indirect consequence of incomplete spermatocyte maturation [33], [34]. *bol* seems to function at a step after *ofs*, controlling the onset of metaphase I by activating *twe* mRNA translation. In *bol* mutants Twe is not expressed and meiotic progression is blocked because CDC2 remains phosphorylated and inactive. *orb2* mutations have a very different effect on Twe. First, Twe is precociously expressed in cysts containing spermatocytes that have not fully matured. Second, very high levels of Twe accumulate in mature cysts that are arrested prior to metaphase I. Moreover, as would be expected, a substantial fraction of Cdc2 in *orb2* testes is dephosphorylated. Finally, Twe persists in differentiating spermatids. These phenotypes, together with the high levels of the A and B Cyclins, argue that *orb2* regulates meiotic progression at a step that is likely later than either *ofs* or *bol*. Additionally, these findings indicate that meiotic progression in male flies does not depend upon a single critical step or “switch” such as turning on *twe* or *cyclin* mRNA translation. Rather, it would appear that multiple steps in meiotic progression are subject to translational regulation, and that these steps are controlled by different translation factors.

One simple model for Twe (Twe-LacZ) misexpression is that *orb2* represses the translation of *twe* mRNA, perhaps by antagonizing Bol dependent activation. However, there are complications with this model. For example, the high levels of Twe-LacZ that accumulate in cysts arrested before metaphase I could be the consequence of a prolonged arrest at a point after Bol activation of *twe* translation rather than a failure to repress *twe* mRNA translation. While an indirect effect of this type would not explain why Twe-LacZ is precociously expressed in immature *orb2* spermatocytes, we were unable to demonstrate an association between Orb2 and *twe* mRNA. Additionally, *twe* 3′ UTR doesn't contain any obvious CPE-like recognition sequences. With the caveat that these are negative results, an alternative possibility is that the effects on Twe-LacZ expression are indirect.

The onset of spermatid differentiation in wild type normally proceeds only after the completion of meiosis. However, as is seen for *twe*, *ofs* and *bol*, differentiation becomes uncoupled from meiotic progression and the mutant cysts ultimately exit the pre-metaphase I arrest and begin the process of spermatid differentiation [32]–[34]. In all of these mutants the differentiation process is abnormal, with some steps being initiated, but not properly executed, while other steps are not even initiated. One of the key events in spermatid differentiation is the elongation of the flagellar axoneme. Little or no elongation is evident for *bol*, while *twe* and *ofs* spermatids begin elongating but quickly abort [32]–[34]. While the spermatid flagellar axonemes elongate in *orb2* mutants, the axonemes don't extend straight back towards the stem cells at the tip of the testes, but instead zigzag irregularly and prematurely halt elongation. They also have an abnormal internal morphology and though they express Bol, they lack the prominent Bol band, which in wild type testes co-localizes with the Orb2 band near the tip of the elongating axonemes. Since Bol is essential for elongation, the absence of the Bol band is likely to be relevant to the elongation defects in *orb2*. While we didn't detect any association between Orb2 and *bol* mRNA, RNA independent Orb2-Bol proteins complexes are found in testes extracts. Thus, a plausible idea is that localization of Bol to the axonemal band is mediated by interactions with Orb2.

Once elongation is completed in wild type, the spermatid nuclei condense and coalesce into a nuclear bundle and this structure provides a scaffold for assembling the IC. In *orb2* the spermatid nuclei don't properly condense and never coalesce into a tight nuclear bundle. Though the process of IC assembly is initiated and actin cones are generated, a complete IC is never formed. The individualization marker Don Juan is also not expressed in *orb2* testes. Interestingly, though spermatid differentiation appears to be much less complete in *ofs* than in *orb2*, Don Juan is expressed in *ofs* testes [33].

An important question is whether the defects in differentiation evident in *orb2* testes reflect functions for *orb2* during this stage of spermatogenesis or are the indirect and perhaps non-specific consequence of the earlier meiotic arrest. Arguing against the later possibility is the fact that *ofs*, *bol*, and *orb2* mutants have quite distinct differentiation phenotypes, yet all three fail to undergo meiosis. In the case of *orb2*, other lines of evidence point to functions at specific steps in differentiation. First, *orb2* appears to be required for repressing the post-meiotic expression of Orb until after spermatid elongation is complete. In wild type, the *orb* gene is transcribed post-meiotically, but *orb* mRNA is not translated until after spermatid elongation is nearly complete. Since the timing of *orb* mRNA translation is correlated with the disappearance of Orb2, a plausible idea is that Orb2 represses *orb* mRNA translation. Consistent with this hypothesis, the levels of Orb protein are elevated in *orb2* mutant testes, and it is expressed prematurely in incompletely elongated spermatids. In addition, instead of being expressed only at the tip of the flagellar axonemes, Orb is distributed all along the axonemes. As *orb* mRNA contains two CPE elements and can be detected readily in Orb2 immunoprecipitates, it seems possible that Orb2 could directly repress *orb* mRNA translation. As noted above, a role in repressing *orb* mRNA translation is also suggested by genetic interaction studies in females (Nathaniel Hafer, PhD thesis). Second, *orb* mRNA does not seem to be the only post-meiotic *orb2* regulatory target. We found that *scotti* and *f-cup*, which are also expressed after meiosis and thought to encode proteins involved in differentiation, are found in Orb2 immunoprecipitates of testes extracts. Moreover, there could be additional targets besides these three mRNAs. Several of the other post-meiotically expressed genes have CPE-like elements in their 3′ UTRs [26]. Similarly, the mRNA encoding *gld2* poly(A) polymerase, which is thought to be an Orb co-factor, also has a CPE-like element in its 3′ UTR and resembles Orb in that Gld2 protein preferentially accumulates near the tip of elongated flagellar axonemes [51]. Third, the hypomorphic poly Q deletion mutant, *orb2^ΔQ^*, makes it possible to separate meiotic arrest from at least some steps in differentiation. Meiosis appears to be completely unaffected by the *ΔQ* mutation; however, as is seen for *orb2^36^* there are defects in both flagellar axoneme elongation and IC assembly. On the other hand, since the differentiation defects in *orb2^ΔQ^* are much less severe than those in the null, the possibility remains open that the failure in meiosis interferes with some process(es) critical for proper differentiation. For example, the defects in chromosome condensation and spermatid nuclear bundle formation could be due to the fact that the *orb2* spermatid nuclei have a large excess of DNA. In turn it could be argued that the failure in IC assembly in *orb2* is due to the absence of a coalesced spermatid nuclear bundle. However, the fact that IC assembly is also defective in *orb2* ^ΔQ^ would argue that *orb2* must have IC specific functions that are independent of any IC assembly steps that require completion of meiosis. Consistent with this possibility, mRNAs encoding Scotti, which has also been implicated in IC function, are found in *orb2* immunoprecipitates.

Finally, our studies provide some insights into the functional properties of the N-terminal region of the Orb2 protein. First, the very modest phenotypes observed not only in the soma [14], [19] but also in the male germline for *orb2^ΔQ^* suggest that the prion forming poly-Q domain, which is present in both the 75 kD and the 60 kD isoforms [10], [11], is dispensable for most *orb2* functions. Second, even though the testes differ from the soma in that there are readily detectable levels of the 60 kD isoform, it is not clear what function if any this isoform has in spermatogenesis. In the insertion mutants that reduce expression of the 75 kD isoform there are even higher levels than normal of the 60 kD isoform, yet these mutants exhibit meiotic and differentiation defects that resemble those seen for the *orb2* deletions. Though their phenotypes appear less severe than the deletion mutants, this could be attributed to the fact that all express some residual 75 kD protein. Third, the 162 N-terminal sequence that is unique to the 75 kD isoform is critical for *orb2* function in programming the orderly development of the male germline from meiosis through the process of spermatid differentiation. Since there is little if any of the 60 kD isoform in somatic tissues, it isn't certain at this point whether the smaller isoform would be able substitute for the 75 kD in the soma. Additional tools will be required to determine whether the smaller isoform has any role in spermatogenesis and also to further dissect how *orb2* functions at different points in meiosis and differentiation.

## Materials and Methods

### Fly strains

We obtained P-element/Piggybac insertion (f01556, c06090, e01925, d01793, f04965) from the Exelexis collection maintained at Harvard [29]. Deficiency stocks Df(3L)ED4421, Df(3L)ED4415, Df(3L)ED4416 and the *dj*-GFP stock were obtained from the *Drosophila* stock center (Bloomington). *bol^1^* is a kind gift from Steven Wasserman [37]. *orb2^Δ^* and *orb2^ΔQ^* are provided by Barry Dickson [14]. All *twine-lacZ* flies, *twine*, *aly*, *sa*, *can*, and *mia* mutants are kindly provided by Minx Fuller (Stanford).

### Fertility assay

20 individual males were placed with two *w^1118^* females each in food vials for 5 days, after which adults were removed. Presence of larvae, pupae and adults were examined after another 2 weeks. Those with presence of larvae are considered fertile.

### Generating *orb2* null allele

*piggyBack (pBac)* transposon insertions with FRT sites near the *orb2* gene used to generate *orb2* null alleles are: FRT1 (f01556), FRT2 (d01925), FRT3 (f04965) (Figure S2). The FRT sites are used in combination with FLP to create targeted deletions of genomic DNA (method as described in [29], [30]). Deletions were confirmed using PCR primers specific for *pBac* sequences flanking the deletion site and within the gene region. We recovered and established several independent deletion stocks from each transposon pair and they behave similarly. Experiments described here use deletions from f01556–f04965, which we named *orb2^36^*. There are also deficiencies in the region that uncover the *orb2* locus and have been mapped molecularly (Df(3L)ED4421, Df(3L)ED4415, Df(3L)ED4416). They behave the same when combined with *orb2^36^*. In the text, Df(3L)ED4416 is used and referred to as 4416.

### Western blotting

Western blotting was essentially performed as in [19]. Antibodies used were as follows: mouse anti-Orb2 2D11 (1∶25), mouse anti-Orb2 4G8 (1∶25), mouse anti-Snf 4G3 (1∶2000), rabbit anti CDC2 (PSTAIR) (1∶2000, millipore), rabbit anti CDC2^Tyr15^ (IMG668) (1∶2000, IMGENEX), mouse anti-Orb 6H4 (1∶60), mouse anti-Orb 4H8 (1∶60) [3], [4], goat anti-mouse conjugated HRP (1∶1000- Jackson Immunoresearch). Blots were then washed 4×10 minutes in TBST and developed with ECL-plus (Amersham).

### *In situ* hybridization

*In situ* hybridization was performed as described in [52]. Fluorescent antisense probes for *orb2* were synthesized by Biosearch Technologies (www.biosearchtech.com). Forty non-overlapping 17 bp probes targeted at *orb2* mRNA sequence from cctggacgatcagatgt to atatgttatttaatctcac were synthesized and labeled with Quasar 670 and used at 1∶100 dilution. Detection is done on an inverted Zeiss LSM510 confocal microscope.

### Immunocytochemistry

Whole mount staining is performed as in [33]. Antibodies used were as follows: mouse anti-Orb2 2D11 and 4G8 IgG (undiluted), rabbit anti-Bol (1∶1000, a gift from Steven Wasserman), mouse anti-Myosin VI 3C7 1∶25 (a gift from Kathryn Miller), monoclonal anti-β-Tubulin E7 1∶50 (Developmental Studies Hybridoma Bank), monoclonal anti-Orb 6H4 and 4H8 1∶30. DNA was stained with Hoescht (1∶1000). Actin was stained with Alexa488-phalloidin, Alexa546-phalloidin (Invitrogen, Carlsbad, CA). Secondary antibodies used were goat anti-mouse IgG Alexa 488 or 546, goat anti-rabbit Alexa 488 or 546 (Molecular Probes, Inc.) Samples were mounted in Aqua-polymount on slides for an inverted Zeiss LSM510 confocal microscope. Testes live squash and phase contrast was performed as described in [41]. β-galactosidase activity assay was performed as described by [28].

### Immunoprecipitation and RT–PCR

Immunoprecipitation was performed essentially as described by [53], except the followings: crude monoclonal anti-Orb2 antibodies 2D11 and 4G8 were affinity purified with Orb2 coupled HiTrap NHS-activated HP column (GE healthcare) before used for immunoprecipitation; purified Orb2 antibodies were mixed with testis extract for 0.5 h–2 h at room temperature before protein-A/G beads (Calbiochem/Millipore) were added in; the mixture was then incubated at 4 C° for 2 h to overnight. Putative Orb2 target mRNAs with CPE binding sites were predicted using software described in [54]. RT-PCR was done according to [19].

Primers used were as follows:

*orb2* common exon among RA,B,C,D:

CAACAGTGCCACCAGCAGTGC and GCGCAGACTAACTTCGTCGTT.

*Cg5741*: ATGAGCAAAGCTCCGTTGAAAGCC and TATCCGGATTAACCGTGTTCCGCA.

*orb* :CAAGCCCTTGACTCGCAACTCC and CTCCGCCATATTTCTACGTCGCCTAC

*scotti*: AAGAACCTCTCTTGGACCTCGGAA and AATGGGATGCATATCGGCTGGTTG

*f-cup*: AACCAGCTGAGCACTTTGCCCAAT and AGATGAACTGTGGCACATAGCCGA

### Phosphorylation assay

Phosphorylation assay was done as in [55]. Testis were squashed in cold PBS and treated with λ protein phosphatase for 1 hour at 30°C followed by Western blots.

## Supporting Information

Figure S1 Orb2 is hyperphosphorylated in tTAFs mutant testes. A) Orb2 migrates slower in testes extract from tTAF mutants, but not the head. B) λ-phosphotase (λPP) treatment removes the slow migrating form of Orb2 in *sa^1^* mutant testes, indicating hyperphosphorylation.
(TIF)

Figure S2 *orb2* gene structure, *orb2* mRNA and protein expression in its mutant alleles. A) *orb2* gene structure adapted from Flybase. *orb2* has multiple transcripts. RA, RB, RC and RD are CPEB homologs that are transcribed from three different promoters (blue arrow, *orb2-1*, *2*, *3*). There is another transcript RH not shown here that shares the same RC sequence with a larger 3′UTR). RE, RF, RG and RI are fusion transcripts of sequences from the 5′ most exons of *orb2* and a downstream gene, *CG5741*. These chimeric RNAs (*orb2-CG5741*) encode part of the Orb2 N-terminal domain, but do not have the conserved CPEB homology domain. *CG5741* has its own promoter, and normal levels of *CG5741* transcripts are observed in the various *orb2* mutants [19]. In contrast, alterations in the levels of the various *orb2-CG5741* chimeric RNAs are observed in different *orb2* insertion mutants. *piggyBac* insertion sites are marked by black arrows. *piggyBac 1556*, *1925* and *4965* contain properly oriented FRT sites (FRT1, 2, 3) for generating deletion alleles through mitotic recombination (inset shows schemes of generating deletion from two adjacent FRT sites [29], [30]). Red brackets mark the poly-Q sequence that is deleted in *orb2^ΔQ^* allele [14]. B) *6090* and *1925* insertion disrupts Orb2 expression in the testes and the heads. The 1793 insertion, on the other hand, only affects Orb2 expression in the testes (suggesting that *orb2-2* is active in the head, while most but not all of the transcripts in the test are from *orb2-1*). Snf is used as a loading control. C) Effects of *piggyBac* insertion on *orb2* and *orb2-CG5741* transcripts. Notice that *4965* insertion disrupts *orb2-CG5741* but has no effect on *orb2* transcripts. It also has no effect on the levels of *CG5741* RNAs. *4965* has no spermatogenesis defects, indicating that the spermatogenesis phenotype we saw is a result of loss of Orb2 function (see further discussion in [19]). *6090* ^−1^, which precisely removes *6090 piggyBac* insertion, fully restores *orb2* transcript, protein expression and fertility.
(TIF)

Figure S3 Bol expression in spermatocytes and spermatids in *orb2^36^*. A, B, C, D: Bol antibody staining; A′, B′, C′, D′, Bol (green) and DNA (blue) overlay. A, A′) Biphasic subcellular localization of Bol in wild type testes. Bol is seen both in the cytoplasm (arrow) and in a perinucleus dot (arrowhead) in spermatocytes in wild type testes. B, B′) This pattern is also observed in *orb2^36^*. C, C′, D, D′) Bol cytoplasmic localization in spermatids is observed in wild type spermatids and pseudo spermatids in *orb2^36^*. Scale bar: 50 µm.
(TIF)

Figure S4 “comet” and “cup” classes of genes are detected in anti-Orb2 immunoprecipitates. mRNAs isolated from anti-Orb2 immunoprecipitates were reversed transcribed with oligo-dT primers. cDNAs were then PCR amplified using primers derived from the 3′UTRs of *scotti* and *f-cup*. Both genes are expressed post-meiotically and encode mRNAs with CPEs in their 3′ UTR. *orb2^36^* testes extract is used as a negative control for non-specific immunoprecipitation.
(TIF)

## References

[1] Christerson LB, McKearin DM (1994) *orb* is required for anteroposterior and dorsoventral patterning during *Drosophila* oogenesis. Genes Dev 8: 614–628. 7926753 10.1101/gad.8.5.614

[2] Hake LE, Richter JD (1994) CPEB is a specificity factor that mediates cytoplasmic polyadenylation during *Xenopus* oocyte maturation. Cell 79: 617–627. 7954828 10.1016/0092-8674(94)90547-9

[3] Lantz V, Ambrosio L, Schedl P (1992) The *Drosophila orb* gene is predicted to encode sex-specific germline RNA-binding proteins and has localized transcripts in ovaries and early embryos. Development 115: 75–88. 1638994 10.1242/dev.115.1.75

[4] Lantz V, Chang JS, Horabin JI, Bopp D, Schedl P (1994) The *Drosophila orb* RNA-binding protein is required for the formation of the egg chamber and establishment of polarity. Genes Dev 8: 598–613. 7523244 10.1101/gad.8.5.598

[5] Groisman I, Jung MY, Sarkissian M, Cao Q, Richter JD (2002) Translational control of the embryonic cell cycle. Cell 109: 473–483. 12086604 10.1016/s0092-8674(02)00733-x

[6] Novoa I, Gallego J, Ferreira PG, Mendez R (2010) Mitotic cell-cycle progression is regulated by CPEB1 and CPEB4-dependent translational control. Nat Cell Biol 12: 447–456. 20364142 10.1038/ncb2046

[7] Burns DM, D'Ambrogio A, Nottrott S, Richter JD (2011) CPEB and two poly(A) polymerases control miR-122 stability and p53 mRNA translation. Nature 473: 105–108. 21478871 10.1038/nature09908 PMC3088779

[8] Burns DM, Richter JD (2008) CPEB regulation of human cellular senescence, energy metabolism, and p53 mRNA translation. Genes Dev 22: 3449–3460. 19141477 10.1101/gad.1697808 PMC2607074

[9] Wu L, Wells D, Tay J, Mendis D, Abbott MA, et al (1998) CPEB-mediated cytoplasmic polyadenylation and the regulation of experience-dependent translation of alpha-CaMKII mRNA at synapses. Neuron 21: 1129–1139. 9856468 10.1016/s0896-6273(00)80630-3

[10] Si K, Choi YB, White-Grindley E, Majumdar A, Kandel ER (2010) *Aplysia* CPEB can form prion-like multimers in sensory neurons that contribute to long-term facilitation. Cell 140: 421–435. 20144764 10.1016/j.cell.2010.01.008

[11] Si K, Lindquist S, Kandel ER (2003) A neuronal isoform of the *Aplysia* CPEB has prion-like properties. Cell 115: 879–891. 14697205 10.1016/s0092-8674(03)01020-1

[12] Luitjens C, Gallegos M, Kraemer B, Kimble J, Wickens M (2000) CPEB proteins control two key steps in spermatogenesis in C. elegans. Genes Dev 14: 2596–2609. 11040214 10.1101/gad.831700 PMC316992

[13] Mendez R, Richter JD (2001) Translational control by CPEB: a means to the end. Nat Rev Mol Cell Biol 2: 521–529. 11433366 10.1038/35080081

[14] Keleman K, Kruttner S, Alenius M, Dickson BJ (2007) Function of the *Drosophila* CPEB protein Orb2 in long-term courtship memory. Nat Neurosci 10: 1587–1593. 17965711 10.1038/nn1996

[15] Chang JS, Tan L, Schedl P (1999) The *Drosophila* CPEB homolog, orb, is required for oskar protein expression in oocytes. Dev Biol 215: 91–106. 10525352 10.1006/dbio.1999.9444

[16] Chang JS, Tan L, Wolf MR, Schedl P (2001) Functioning of the *Drosophila orb* gene in gurken mRNA localization and translation. Development 128: 3169–3177. 11688565 10.1242/dev.128.16.3169

[17] Tan L, Chang JS, Costa A, Schedl P (2001) An autoregulatory feedback loop directs the localized expression of the *Drosophila* CPEB protein Orb in the developing oocyte. Development 128: 1159–1169. 11245581 10.1242/dev.128.7.1159

[18] Castagnetti S, Ephrussi A (2003) Orb and a long poly(A) tail are required for efficient oskar translation at the posterior pole of the *Drosophila* oocyte. Development 130: 835–843. 12538512 10.1242/dev.00309

[19] Hafer N, Xu S, Bhat KM, Schedl P (2011) The *Drosophila* CPEB protein Orb2 has a novel expression pattern and is important for asymmetric cell division and nervous system function. Genetics 189: 907–921. 21900268 10.1534/genetics.110.123646 PMC3213381

[20] Mastushita-Sakai T, White-Grindley E, Samuelson J, Seidel C, Si K (2010) *Drosophila* Orb2 targets genes involved in neuronal growth, synapse formation, and protein turnover. Proc Natl Acad Sci U S A 107: 11987–11992. 20547833 10.1073/pnas.1004433107 PMC2900709

[21] Majumdar A, Cesario WC, White-Grindley E, Jiang H, Ren F, et al (2012) Critical role of amyloid-like oligomers of *Drosophila* Orb2 in the persistence of memory. Cell 148: 515–529. 22284910 10.1016/j.cell.2012.01.004

[22] Fuller MT (1998) Genetic control of cell proliferation and differentiation in *Drosophila* spermatogenesis. Semin Cell Dev Biol 9: 433–444. 9813190 10.1006/scdb.1998.0227

[23] Fuller MT (1993). Spermatogenesis. In The Development of Drosophila (ed. M. Bate and A. Martinez-Arias), pp. 71–147. Cold Spring Harbor, New York: Cold Spring Harbor Press.

[24] Santel A, Blumer N, Kampfer M, Renkawitz-Pohl R (1998) Flagellar mitochondrial association of the male-specific Don Juan protein in *Drosophila* spermatozoa. J Cell Sci 111 (Pt 22) 3299–3309. 9788872 10.1242/jcs.111.22.3299

[25] Hempel LU, Rathke C, Raja SJ, Renkawitz-Pohl R (2006) In *Drosophila*, don juan and don juan like encode proteins of the spermatid nucleus and the flagellum and both are regulated at the transcriptional level by the TAF II80 cannonball while translational repression is achieved by distinct elements. Dev Dyn 235: 1053–1064. 16477641 10.1002/dvdy.20698

[26] Barreau C, Benson E, Gudmannsdottir E, Newton F, White-Cooper H (2008) Post-meiotic transcription in *Drosophila* testes. Development 135: 1897–1902. 18434411 10.1242/dev.021949

[27] Hiller M, Chen X, Pringle MJ, Suchorolski M, Sancak Y, et al (2004) Testis-specific TAF homologs collaborate to control a tissue-specific transcription program. Development 131: 5297–5308. 15456720 10.1242/dev.01314

[28] White-Cooper H, Schafer MA, Alphey LS, Fuller MT (1998) Transcriptional and post-transcriptional control mechanisms coordinate the onset of spermatid differentiation with meiosis I in *Drosophila* . Development 125: 125–134. 9389670 10.1242/dev.125.1.125

[29] Parks AL, Cook KR, Belvin M, Dompe NA, Fawcett R, et al (2004) Systematic generation of high-resolution deletion coverage of the *Drosophila melanogaster* genome. Nat Genet 36: 288–292. 14981519 10.1038/ng1312

[30] Thibault ST, Singer MA, Miyazaki WY, Milash B, Dompe NA, et al (2004) A complementary transposon tool kit for *Drosophila melanogaster* using P and piggyBac. Nat Genet 36: 283–287. 14981521 10.1038/ng1314

[31] Lin TY, Viswanathan S, Wood C, Wilson PG, Wolf N, et al (1996) Coordinate developmental control of the meiotic cell cycle and spermatid differentiation in *Drosophila* males. Development 122: 1331–1341. 8620860 10.1242/dev.122.4.1331

[32] Eberhart CG, Maines JZ, Wasserman SA (1996) Meiotic cell cycle requirement for a fly homologue of human Deleted in Azoospermia. Nature 381: 783–785. 8657280 10.1038/381783a0

[33] Baker CC, Fuller MT (2007) Translational control of meiotic cell cycle progression and spermatid differentiation in male germ cells by a novel eIF4G homolog. Development 134: 2863–2869. 17611220 10.1242/dev.003764 PMC4620998

[34] Franklin-Dumont TM, Chatterjee C, Wasserman SA, Dinardo S (2007) A novel eIF4G homolog, Off-schedule, couples translational control to meiosis and differentiation in *Drosophila* spermatocytes. Development 134: 2851–2861. 17611222 10.1242/dev.003517

[35] Cheng MH, Maines JZ, Wasserman SA (1998) Biphasic subcellular localization of the DAZL-related protein boule in *Drosophila* spermatogenesis. Dev Biol 204: 567–576. 9882490 10.1006/dbio.1998.9098

[36] Maines JZ, Wasserman SA (1999) Post-transcriptional regulation of the meiotic Cdc25 protein Twine by the Dazl orthologue Boule. Nat Cell Biol 1: 171–174. 10559904 10.1038/11091

[37] Alphey L, Jimenez J, White-Cooper H, Dawson I, Nurse P, et al (1992) twine, a cdc25 homolog that functions in the male and female germline of *Drosophila* . Cell 69: 977–988. 1606618 10.1016/0092-8674(92)90616-k

[38] Sigrist S, Ried G, Lehner CF (1995) Dmcdc2 kinase is required for both meiotic divisions during *Drosophila* spermatogenesis and is activated by the Twine/cdc25 phosphatase. Mech Dev 53: 247–260. 8562426 10.1016/0925-4773(95)00441-3

[39] Tokuyasu KT, Peacock WJ, Hardy RW (1972) Dynamics of spermiogenesis in *Drosophila melanogaster*. I. Individualization process. Z Zellforsch Mikrosk Anat 124: 479–506. 4622067 10.1007/BF00335253

[40] Noguchi T, Lenartowska M, Miller KG (2006) Myosin VI stabilizes an actin network during *Drosophila* spermatid individualization. Mol Biol Cell 17: 2559–2571. 16571671 10.1091/mbc.E06-01-0031 PMC1474903

[41] Fabrizio JJ, Hime G, Lemmon SK, Bazinet C (1998) Genetic dissection of sperm individualization in *Drosophila melanogaster* . Development 125: 1833–1843. 9550716 10.1242/dev.125.10.1833

[42] Hasegawa E, Karashima T, Sumiyoshi E, Yamamoto M (2006) C. elegans CPB-3 interacts with DAZ-1 and functions in multiple steps of germline development. Dev Biol 295: 689–699. 16678151 10.1016/j.ydbio.2006.04.002

[43] Thompson BE, Bernstein DS, Bachorik JL, Petcherski AG, Wickens M, et al (2005) Dose-dependent control of proliferation and sperm specification by FOG-1/CPEB. Development 132: 3471–3481. 16000383 10.1242/dev.01921 PMC1350643

[44] Barton MK, Kimble J (1990) fog-1, a regulatory gene required for specification of spermatogenesis in the germ line of Caenorhabditis elegans. Genetics 125: 29–39. 2341035 10.1093/genetics/125.1.29 PMC1204007

[45] Hochegger H, Klotzbucher A, Kirk J, Howell M, le Guellec K, et al (2001) New B-type cyclin synthesis is required between meiosis I and II during *Xenopus* oocyte maturation. Development 128: 3795–3807. 11585805 10.1242/dev.128.19.3795

[46] Igea A, Mendez R (2010) Meiosis requires a translational positive loop where CPEB1 ensues its replacement by CPEB4. EMBO J 29: 2182–2193. 20531391 10.1038/emboj.2010.111 PMC2905248

[47] Sarkissian M, Mendez R, Richter JD (2004) Progesterone and insulin stimulation of CPEB-dependent polyadenylation is regulated by Aurora A and glycogen synthase kinase-3. Genes Dev 18: 48–61. 14724178 10.1101/gad.1136004 PMC314275

[48] Kronja I, Orr-Weaver TL (2011) Translational regulation of the cell cycle: when, where, how and why? Philos Trans R Soc Lond B Biol Sci 366: 3638–3652. 22084390 10.1098/rstb.2011.0084 PMC3203463

[49] Richter JD (2007) CPEB: a life in translation. Trends Biochem Sci 32: 279–285. 17481902 10.1016/j.tibs.2007.04.004

[50] Tay J, Richter JD (2001) Germ cell differentiation and synaptonemal complex formation are disrupted in CPEB knockout mice. Dev Cell 1: 201–213. 11702780 10.1016/s1534-5807(01)00025-9

[51] Sartain CV, Cui J, Meisel RP, Wolfner MF (2011) The poly(A) polymerase GLD2 is required for spermatogenesis in Drosophila melanogaster. Development 138: 1619–1629. 21427144 10.1242/dev.059618 PMC3062429

[52] Raj A, van den Bogaard P, Rifkin SA, van Oudenaarden A, Tyagi S (2008) Imaging individual mRNA molecules using multiple singly labeled probes. Nat Methods 5: 877–879. 18806792 10.1038/nmeth.1253 PMC3126653

[53] Costa A, Wang Y, Dockendorff TC, Erdjument-Bromage H, Tempst P, et al (2005) The Drosophila fragile X protein functions as a negative regulator in the orb autoregulatory pathway. Dev Cell 8: 331–342. 15737929 10.1016/j.devcel.2005.01.011

[54] Pique M, Lopez JM, Foissac S, Guigo R, Mendez R (2008) A combinatorial code for CPE-mediated translational control. Cell 132: 434–448. 18267074 10.1016/j.cell.2007.12.038

[55] Wong LC, Costa A, McLeod I, Sarkeshik A, Yates J 3rd, et al (2011) The functioning of the *Drosophila* CPEB protein Orb is regulated by phosphorylation and requires casein kinase 2 activity. PLoS One 6: e24355. 21949709 10.1371/journal.pone.0024355 PMC3176278

