# Supplementary figures and images for: The CPEB Protein Orb2 Has Multiple Functions during Spermatogenesis in Drosophila melanogaster
Source: PLoS Genet. 2012 Nov 29;8(11):e1003079. doi: 10.1371/journal.pgen.1003079 (PMC3510050; doi:10.1371/journal.pgen.1003079)

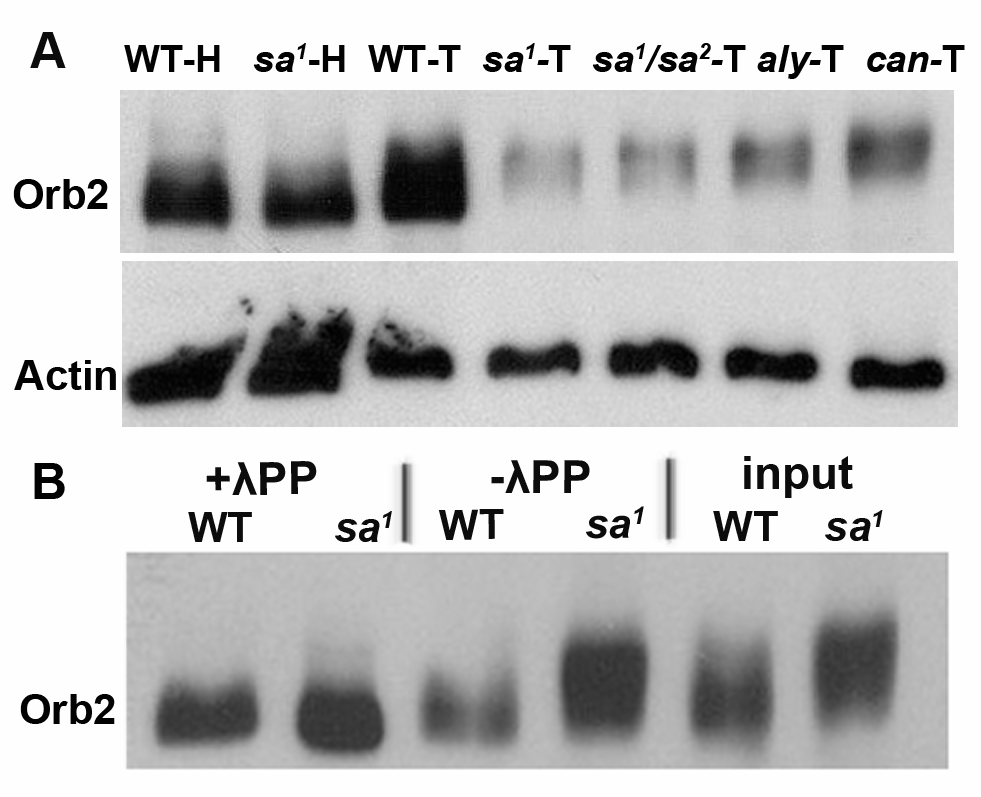

Supplement: Figure S1 — Orb2 is hyperphosphorylated in tTAFs mutant testes. A) Orb2 migrates slower in testes extract from tTAF mutants, but not the head. B) λ-phosphotase (λPP) treatment removes the slow migrating form of Orb2 in sa1 mutant testes, indicating hyperphosphorylation. (TIF) [file pgen.1003079.s001.tif]

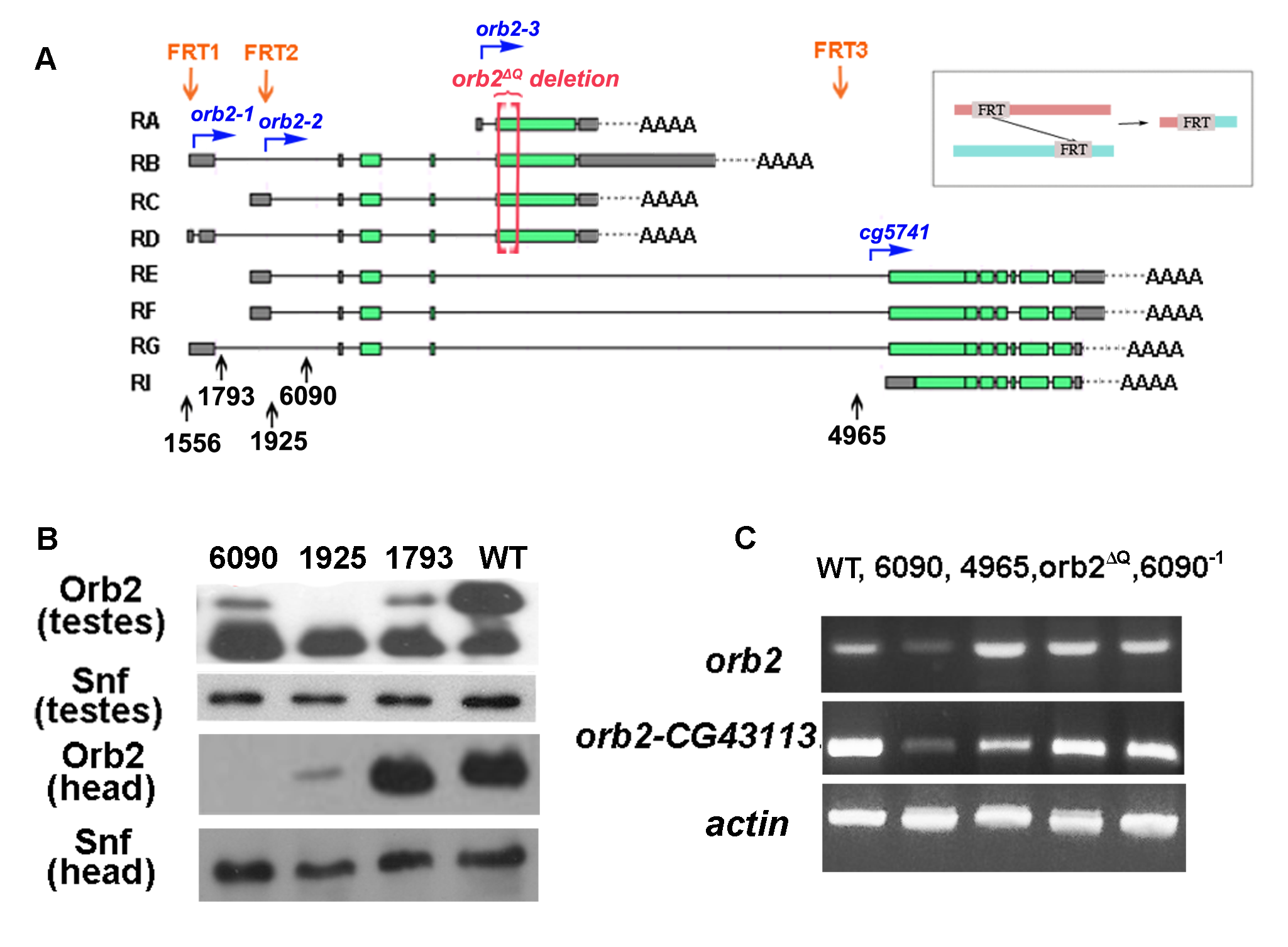

Supplement: Figure S2 — orb2 gene structure, orb2 mRNA and protein expression in its mutant alleles. A) orb2 gene structure adapted from Flybase. orb2 has multiple transcripts. RA, RB, RC and RD are CPEB homologs that are transcribed from three different promoters (blue arrow, orb2-1, 2, 3). There is another transcript RH not shown here that shares the same RC sequence with a larger 3′UTR). RE, RF, RG and RI are fusion transcripts of sequences from the 5′ most exons of orb2 and a downstream gene, CG5741. These chimeric RNAs (orb2-CG5741) encode part of the Orb2 N-terminal domain, but do not have the conserved CPEB homology domain. CG5741 has its own promoter, and normal levels of CG5741 transcripts are observed in the various orb2 mutants [19]. In contrast, alterations in the levels of the various orb2-CG5741 chimeric RNAs are observed in different orb2 insertion mutants. piggyBac insertion sites are marked by black arrows. piggyBac 1556, 1925 and 4965 contain properly oriented FRT sites (FRT1, 2, 3) for generating deletion alleles through mitotic recombination (inset shows schemes of generating deletion from two adjacent FRT sites [29], [30]). Red brackets mark the poly-Q sequence that is deleted in orb2ΔQ allele [14]. B) 6090 and 1925 insertion disrupts Orb2 expression in the testes and the heads. The 1793 insertion, on the other hand, only affects Orb2 expression in the testes (suggesting that orb2-2 is active in the head, while most but not all of the transcripts in the test are from orb2-1). Snf is used as a loading control. C) Effects of piggyBac insertion on orb2 and orb2-CG5741 transcripts. Notice that 4965 insertion disrupts orb2-CG5741 but has no effect on orb2 transcripts. It also has no effect on the levels of CG5741 RNAs. 4965 has no spermatogenesis defects, indicating that the spermatogenesis phenotype we saw is a result of loss of Orb2 function (see further discussion in [19]). 6090 −1, which precisely removes 6090 piggyBac insertion, fully restores orb2 transcript, protein [file pgen.1003079.s002.tif]

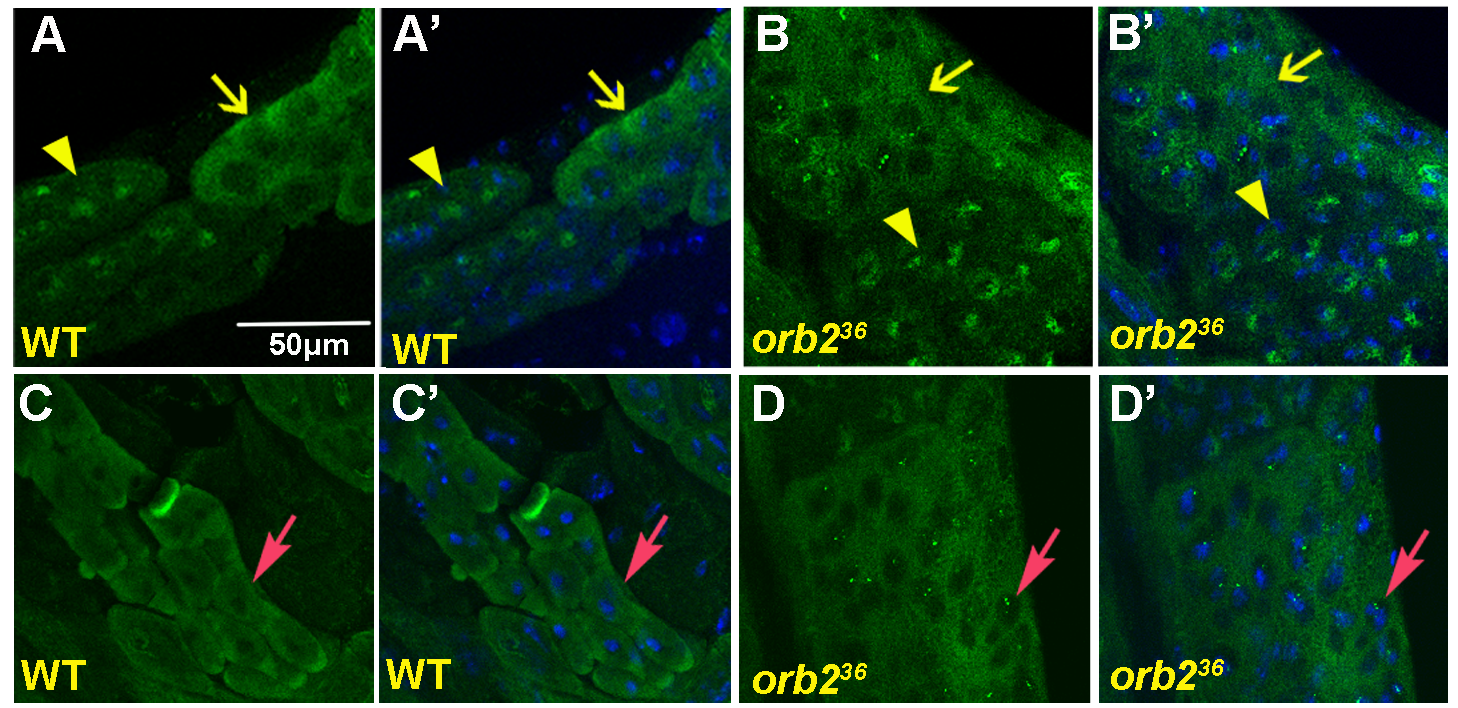

Supplement: Figure S3 — Bol expression in spermatocytes and spermatids in orb236. A, B, C, D: Bol antibody staining; A′, B′, C′, D′, Bol (green) and DNA (blue) overlay. A, A′) Biphasic subcellular localization of Bol in wild type testes. Bol is seen both in the cytoplasm (arrow) and in a perinucleus dot (arrowhead) in spermatocytes in wild type testes. B, B′) This pattern is also observed in orb236. C, C′, D, D′) Bol cytoplasmic localization in spermatids is observed in wild type spermatids and pseudo spermatids in orb236. Scale bar: 50 µm. (TIF) [file pgen.1003079.s003.tif]

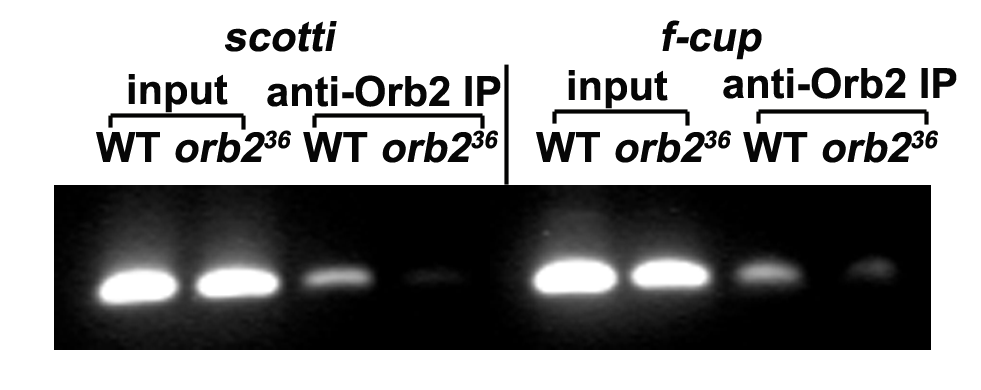

Supplement: Figure S4 — “comet” and “cup” classes of genes are detected in anti-Orb2 immunoprecipitates. mRNAs isolated from anti-Orb2 immunoprecipitates were reversed transcribed with oligo-dT primers. cDNAs were then PCR amplified using primers derived from the 3′UTRs of scotti and f-cup. Both genes are expressed post-meiotically and encode mRNAs with CPEs in their 3′ UTR. orb236 testes extract is used as a negative control for non-specific immunoprecipitation. (TIF) [file pgen.1003079.s004.tif]
